# Characteristics and Impact of U.S. Military Blast-Related Mild Traumatic Brain Injury: A Systematic Review

**DOI:** 10.3389/fneur.2020.559318

**Published:** 2020-11-02

**Authors:** Helen Phipps, Stefania Mondello, Arlington Wilson, Travis Dittmer, Natalie N. Rohde, Paul J. Schroeder, Jaime Nichols, Camille McGirt, Justin Hoffman, Kaila Tanksley, Mariam Chohan, Amanda Heiderman, Hussein Abou Abbass, Firas Kobeissy, Sidney Hinds

**Affiliations:** ^1^Booz Allen Hamilton, San Antonio, TX, United States; ^2^Department of Biomedical and Dental Sciences and Morphofunctional Imaging, University of Messina, Messina, Italy; ^3^Department of Neurology IC, Oasi Research Institute-IRCCS, Troina, Italy; ^4^Dell, Round Rock, TX, United States; ^5^Department of Anatomy, Cell Biology and Physiological Sciences, Faculty of Medicine, American University of Beirut, Beirut, Lebanon; ^6^Department of Psychiatry, Center for Neuroproteomics and Biomarkers Research, University of Florida, Gainesville, FL, United States; ^7^Medical Research and Development Command, Ft Detrick, MD, United States

**Keywords:** blast, mild traumatic brain injury, U.S. military, PTSD, systematic review, epidemiology

## Abstract

As a result of armed conflict, head trauma from exposure to blasts is an increasing critical health issue, particularly among military service members. Whilst numerous studies examined the burden of blast-related brain injuries on service members', few systematic reviews have been published. This work provides a comprehensive summary of the evidence on blast-related mild traumatic brain injury (mTBI) burden in active U.S. military service members and inactive Veterans, describing characteristics and outcomes. Records published up to April 2017 were identified through a search of PubMed, Web of Science, Scopus, Ovid MEDLINE, and Cochrane Library. Records-based and original research reporting on U.S. military service members and Veterans with mild blast TBI were included. Data on subject characteristics, exposure, diagnostic criterion, and outcomes were extracted from included studies using a standardized extraction form and were presented narratively. Of the 2,290 references identified by the search, 106 studies with a total of 37,515 participants met inclusion criteria for blast-related mTBI. All but nine studies were based out of military or Veteran medical facilities. Unsurprisingly, men were over-represented (75–100%). The criteria used to define blast-related mTBI were consistent; however, the methodology used to ascertain whether individuals met those criteria for diagnosis were inconsistent. The diagnosis, most prevalent among the Army, heavily relied on self-reported histories. Commonly reported adverse outcomes included hearing disturbances and headaches. The most frequently associated comorbidities were post-traumatic stress disorder, depression, anxiety, sleep disorders, attention disorders, and cognitive disorders. The primary objective of this review was to provide a summary of descriptive data on blast-related mTBI in a U.S. military population. Low standardization of the methods for reaching diagnosis and problems in the study reporting emphasize the importance to collect high-quality data to fill knowledge gaps pertaining to blast-related mTBI.

## Introduction

Mild traumatic brain injury (mTBI) has been coined as one of the signature wounds of war with blast-related injuries being the most militarily unique (1, 2). Causes of mTBI from blast-related incidents are multifaceted in that the physical wound to the brain may result from direct and/or indirect exposure to over-pressure environments. Improvised explosive devices, occupational training, and heavy munitions firing are common sources of blast injuries incurred during military services (3). Despite more than a decade of research, the etiology, treatment, and recovery from blast-related mTBI remains poorly understood.

The Department of Defense (DoD) defines five mechanisms of blast-related brain injury ranging from primary to quinary and referred to as a taxonomy of injuries from explosive devices as outlined in Table 1 (5). Owing to its unique outcomes, the entire spectrum of all severities and mechanisms of blast-related brain injury is now recognized as a specific area of focus within the broad spectrum of TBI (6). There are several reasons why this development is gaining momentum: (1) its impact on the readiness of militaries worldwide, (2) its coexistence with secondary through quinary effects (7), and (3) the overlapping symptomology with post-traumatic stress disorder (PTSD). Nevertheless, to date, reliable estimates of the burden of blast-related mTBI are lacking, mainly because of ambiguity about the definition among medical practitioners and researchers, absence of objective tests to make a definitive diagnosis of blast-related mTBI especially in an operational environment, and the potential overlap and co-existence of other neuropathological conditions (e.g., PTSD). In fact, it has been shown that depression and PTSD are major components of the psychological changes that accompany mTBI as shown by Hoge et al. (8) and more recently that suicide is associated with mTBI (9). Thus, there is a need for research to fill the gaps in knowledge about blast-related mTBI.

**Table 1:** Taxonomy of injuries from explosive devices per DoD Blast Injury Research Coordinating Office.

**Blast injury category**	**Description**	**Examples**
Primary	Result from the high pressures, or blast overpressure, created by explosions. Blast overpressure can crush the body and cause internal injuries. Primary blast injuries are the only category of blast injuries that are unique to the blast or high pressures that occur.	Blast lung (pulmonary barotrauma) Tympanic Membrane rupture and middle ear damage Abdominal hemorrhage and perforation Globe (eye) rupture Concussion (mild traumatic brain injury (mTBI) without physical signs of head injury)
Secondary	Result when strong blast winds behind the pressure front propel fragments and debris against the body and cause blunt force and penetrating injuries.	Penetrating ballistic (fragmentation or blunt injuries) Eye penetration Closed or open brain injuries
Tertiary	Result from strong blast winds and pressure gradients that can accelerate the body and cause the same types of blunt force injuries that would occur in a car crash, fall, or building collapse.	Bone fractures Traumatic amputations Blunt injuries Crush injuries Closed or open brain injuries
Quaternary	Result from other explosive products (such as heat and light) and from exposure to toxic substances from fuels, metals, and gases that can cause burns, blindness, and inhalation injuries.	Burns (flash, partial, and full thickness) Injury or incapacitation from inhaled toxic fumes (breathing problems from dust, smoke, or toxic fumes)
Quinary	Refer to the clinical consequences of post-detonation environmental contaminants, including chemical (e.g., sarin), biological (e.g., anthrax), and radiological (e.g., dirty bombs) substances.	Chemical burns Radiation exposure Viral or bacterial infections

*Office (4)*.

The direct and indirect costs of the broad spectrum of TBI among Americans, regardless of their military status, were reported as ~$60 billion annually in 2006 (10) and $76.5 billion in 2010 (11). Eibner et al. (12) estimated that per case costs of the full spectrum of deployment-related TBI fell between $96.6 and $144.4 million using a 2007 price level. While this estimated value included hospital care costs, rehabilitation, death, and lost productivity, other economic factors such as substance abuse and homelessness were not considered. Therefore, the reported estimates probably underestimate the “real” economic burden.

Government-sponsored public reports of the broad spectrum of TBI prevalence rates among U.S. military service members are available. For example, the Defense and Veterans Brain Injury Center (DVBIC), a component of the U.S. Military Health System, provides some summary data on prevalence rates. However, publicly available data from the DVBIC are limited to only totals among branches without consideration for multiple TBIs among individual service members and do not provide detailed data on specific types of injuries, such as blast-related mTBI. They only report the highest severity TBI sustained per service member which results in underreporting of TBIs (13). Currently the DVBIC assessment is the best publicly available estimate of U.S. military TBI. More specific inquiries require a formal request to the center. According to the DVBIC, the majority of brain injuries are diagnosed in non-deployed settings (14). Other available data focus on the health outcomes of blast-related mTBI among active U.S. military service members and inactive Veterans (15–19). Although assessments of the cost of TBI on military services have been conducted (20), few systematic assessments of the body of evidence surrounding the prevalence and cost of blast-related mTBI among active U.S. military service members and inactive Veterans have been performed. Additional parameters that can only be obtained from a thorough patient record review and expert characterization and analysis of the actual blast event(s) would be needed for the most accurate assessment of the mechanisms causing the physical injury coupled with the alterations in physiology and pathophysiology. These parameters are beyond the scope of most database/diagnosis-centric resources.

Current estimates from DVBIC indicate that between 2000 and 2019, the total number of service members diagnosed with a TBI was 413,858 (21). This value is congruent with prior reports (22, 23). Although exact current estimates of the prevalence of blast-related mTBI are scarce, evidence from records-based studies offer insight into the scope of the issue. For example, one study (24) reported that, between 2006 and 2011, 70% of the 43,852 patients screened for TBI had a blast-related injury. It should be noted that accurate prevalence and incidence rates of service-related injuries are difficult to ascertain. There are several reasons for this: (1) military service members may under-report injuries (25, 26), (2) medical data may have been entered after the injury incident and/or may be incomplete, (3) mTBIs may be misdiagnosed due to overlapping symptoms with other conditions [e.g., PTSD or “breacher's brain”; (27–29)], (4) differences in how data were analyzed (30), and (5) the reported estimate may be based on data gathered from specific populations (23). Thus, prevalence rates of mTBI among military service members should be interpreted with caution.

The objectives of this study were to review the literature on blast-related mTBI in active U.S. military service members and inactive Veteran populations with the aim of *characterizing the epidemiological patterns of blast-related mTBI* and *assess clinical outcomes*. More specifically, the goal was to examine trends in reported outcomes from a carefully selected group of published reports on blast-related mTBI. The outcomes of interest included:

- Diagnostic and assessment procedures
- Mechanisms of injury
- Inclusion criteria in the study (e.g., active duty and veteran U.S. military)
- Research design (inclusion of a comparative group)
- Time between injury and study participation
- Service branches of the participants
- Demographics of the participants
- Injury associated with TBI (e.g., cognitive and neurosensory impairments, headaches, etc.)
- Comorbidities
- Brain regions of interest (e.g., white matter, cerebrum, thalamus, etc.)
- The financial and work-related cost of treating injuries as reported by the included studies and/or other referenced sources.

For this systematic review the focus is on the general impact of blast-related mTBI, rather than a concentration on acute and post-acute injuries. Veterans were considered inactive consistent with the legal definition according to Title 38 of the Code of Federal Regulations. Primary outcomes of interest were prevalence and incidence of blast-related mTBI in active U.S. military service members and inactive Veterans. Secondary outcomes of interest were related clinical outcomes and economic costs as well as their patterns and changes over time (i.e., study period). The conclusions drawn from this review are necessary to inform research for prevention, resource allocation, and care of blast-related mTBI.

## Materials and Methods

### Design

In accordance with the published protocol (PROSPERO CRD42017054942), a systematic review of studies reporting epidemiological patterns, burden, and outcomes of blast-related mTBI in active U.S. military service members and inactive Veterans was performed. The current systematic review followed guidelines for the Preferred Reporting Items for Systematic Reviews and Meta-Analyses (PRISMA) (31) and the Cochrane Handbook (32).

### Search Strategy

For this systematic review, blast-related mTBI was defined as any mTBI associated with a blast-related event (e.g., over-pressurization impulse, forced air-flow) and included primary, secondary, tertiary, quaternary, and quinary mechanisms (5, 33, 34). Since the population under consideration was limited to U.S. military it was assumed that a diagnosis of mTBI was consistent with the current Department of Defense (DoD), American Congress of Rehabilitation Medicine (ACRM), or the World Health Organization (WHO) guidance, at the time of diagnosis (Table 2). Consistent with DoD guidance (39), articles that reported the individual and cumulative effects of blast components (e.g., primary, secondary, and other damage associated with overpressure wave, maximal pressure, impacts with debris, or ground) were included in this review.

**Table 2:** TBI definitions and diagnostic criteria.

**Organization**	**TBI definition**	**mTBI diagnostic criteria**
DoD/VA (35) (36)	A traumatically induced structural injury and/or physiological disruption of brain function as a result of an external force that is indicated by new onset or worsening of at least one of the following clinical signs, immediately following the event: (1) any period of loss of or a decreased level of consciousness (LOC); (2) any loss of memory for events immediately before or after the injury [post-traumatic amnesia (PTA)]; (3) any alteration in mental state at the time of the injury (e.g., confusion, disorientation, slowed thinking, etc.); (4) neurological deficits^*^ (weakness, loss of balance, change in vision, praxis, paresis/plegia, sensory loss, aphasia, etc.) that may or may not be transient.	Imaging: Normal^*^ LOC: 0–30 min AOC: <24 h PTA: <24 h GCS: 13–15 within 24 h^**^
ACRM (37)	A traumatically induced physiological disruption of brain function, as manifested by at least one of the following: (1) any period of loss of consciousness; (2) any loss of memory for events immediately before or after the accident; (3) any alteration in mental state at the time of the accident (e.g., feeling dazed, disoriented, or confused); and (4) focal neurological deficit(s) that may or may not be transient; but where the severity of the injury does not exceed the following: LOC of ~30 min or less; after 30 min, an initial Glasgow Coma Scale (GCS) of 13–15; and PTA not >24 h.	LOC: 0–30 min AOC: <24 h PTA: <24 h GCS: 13–15 after 30 min
WHO (38)	An acute brain injury resulting from mechanical energy to the head from external physical forces. Operational criteria for clinical identification include: (1) 1 or more of the following: confusion or disorientation, LOC for 30 min or less, post-traumatic amnesia for <24 h, and/or other transient neurologic abnormalities such as focal signs, seizure, and intracranial lesion not requiring surgery; (2) GCS score of 13–15 after 30 min post-injury or later upon presentation for health care; (3) These manifestations of MTBI must not be due to drugs, alcohol, medications, caused by other injuries or treatment for other injuries (e.g., systemic injuries, facial injuries, or intubation), caused by other problems (e.g., psychological trauma, language barrier, or coexisting medical conditions), or caused by penetrating craniocerebral injury.	LOC: 0–30 min AOC: <24 h PTA: <24 h GCS: 13–15 after 30 min

* Removed from and/or not indicated for mTBI in the updated DoD definition.

** *No longer recommended by the DoD as a TBI diagnostic criteria*.

PubMed, Web of Science, Scopus, Ovid MEDLINE, and Cochrane Library databases were systematically searched from their inception dates to April 2017. With the help of an information specialist, search strategies specific to each database were developed using a combination of broad keywords and subject headings/indexing terms. The full search strategies are provided as Supplemental Material.

### Study Selection and Eligibility Criteria

**Figure 1:**
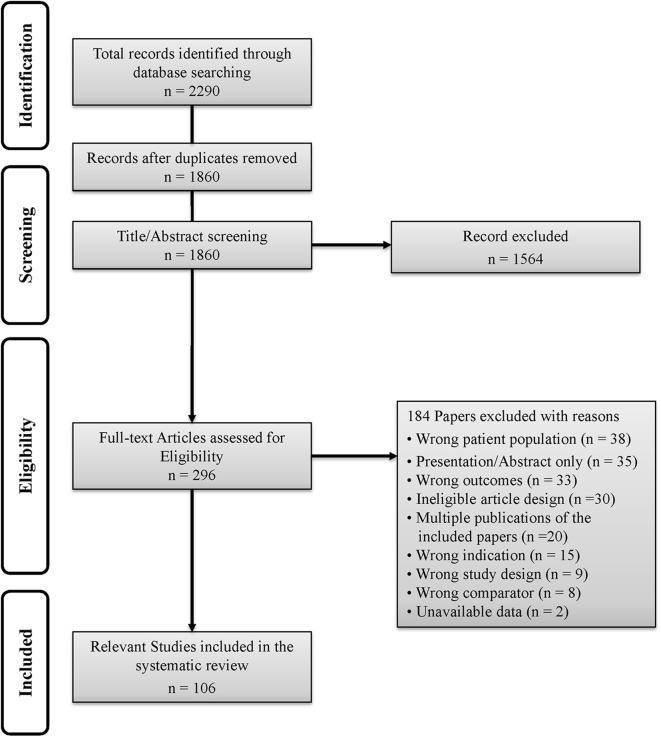
PRISMA flowchart.

The results from the search strategy yielded a total of 2,990 citations, duplicate records were identified and removed (Figure 1). Titles and abstracts of the identified records were screened for relevance. Full text articles of all potentially eligible studies were obtained and evaluated against the inclusion criteria. Studies that were included met the following criteria: (a) the study population was active U.S. military service members and/or inactive Veterans, (b) condition under study included blast-related mTBI and the blast-related mTBI population could be stratified out of other populations included, (c) prevalence, incidence, and/or health outcomes of blast-related mTBI were investigated, and (d) an appropriate study design was used as determined by quality assessment (approach explained below). We excluded studies that: (a) presented mixed populations with a preponderance of moderate or severe blast-related TBI cases that could not be stratified out of the blast-related mTBI cases; and/or (b) condition under study was non-blast TBI (e.g., traditional mechanism TBI).

All languages were included in the searches. Studies with overlapping populations were identified by examining the methodology of each study. Particular attention was paid to the source of the data (e.g., health records, registries, and/or primary research), the time period of the data gathering (if reported), and the location of the data gathering (if reported). Endnote (version X7.5, Thomson Reuters, Toronto, Canada) and a web-based systematic review workflow platform (Covidence, Alfred Health Melbourne, Australia) were used to manage citations.

### Data Extraction

Data extraction was performed by at least two reviewers independently using a standardized (i.e., each reviewer used the same extraction form format and associated data element definitions) and piloted extraction form (i.e., the extraction form was beta tested by several reviewers prior to implementing). Discrepancies were resolved through discussion or arbitration by a third reviewer. Extracted data included information about the study design, setting, population characteristics, case ascertainment methods, TBI assessment and diagnostic modalities, diagnostic criteria, number of blast-related mTBI cases and population denominator (Total), and outcomes.

Studies were stratified by three case ascertainment methods: (1) Medical Records, (2) Original Assessments, or (3) Both. Original assessments included Self-Report, Clinical Assessments, and Imaging as defined below that were administered by the study team. Studies were further analyzed to determine the specific TBI assessment and diagnostic modalities used by researchers. These were categorized as Self-Report, Clinical Assessment, Medical Records, and Imaging as defined below.

- **Self-Report**: Interview or questionnaire using self-reported information
- **Clinical Assessment**: Clinical exams, tests, and neurocognitive assessments performed by a clinician
- **Imaging**: Neuroimaging studies (e.g., MRI or CT)
- **Medical Records**: Data from historical medical records, medical record reviews, or general reference to medical records or medical history.

Study settings were grouped as Military, Civilian, VA, or Combination based on the affiliation of the study centers as described below.

- **Military**: Military treatment facility, field hospital, or research facility
- **Civilian**: Civilian medical or research facility
- **VA**: Veterans Affairs medical or community center
- **Combination**: Any combination of the above.

### Quality Assessment

The quality of the included studies was assessed according to Weiskopf et al. (40). The guideline provides a 5-item checklist—completeness, correctness, concordance, plausibility, and currency. Two reviewers determined whether each study met the five criteria using five factors. The quality of the included studies is summarized in Table 3.

**Table 3:** Quality assessment of included studies.

**#**	**Study ID**	**Completeness**	**Correctness**	**Concordance**	**Plausibility**	**Currency**
1	Adam et al. (41)	✓	✓	✓	✓	✓
2	Akin and Murnane (42)	✘	✓	✓	✓	NR
3	Barlow-Ogden et al. (43)	✘	✓	✓	✓	✘
4	Bazarian et al. (44)	✘	✓	✓	✓	✘
5	Belanger et al. (45)	✘	✓	✓	✓	✘
6	Belanger et al. (46)	✘	✓	✓	✓	✘
7	Bell et al. (47)	✘	✓	✓	✓	✓
8	Bjork et al. (48)	✘	✘	✓	✓	✓
9	Bolzenius et al. (49)	✘	✓	✓	✓	✘
10	Verfaellie et al. (50)	✘	✓	✓	✓	✓
11	Brenner et al. (51)	✘	✘	✓	✓	✘
12	Callahan et al. (52)	✘	✓	✓	✓	NR
13	Capo-Aponte et al. (53)	✘	✓	✓	✓	✓
14	Capo-Aponte et al. (54)	✓	✓	✓	✓	✓
15	Chen et al. (55)	✘	✓	✓	✓	✓
16	Connelly et al. (24)	✘	✓	✓	✓	NR
17	Cooper et al. (56)	✓	✓	✓	✓	✘
18	Davenport et al. (57)	✘	✓	✓	✓	✓
19	Davenport et al. (57)	✘	✓	✓	✓	NR
20	Davenport et al. (58)	✘	✓	✓	✓	NR
21	de Lanerolle et al. (59)	✘	✓	✓	✓	✓
22	Dretsch et al. (60)	✓	✓	✓	✓	✓
23	Erickson (61)	✓	✓	✓	✓	✘
24	Farrell-Carnahan et al. (62)	✘	✘	✓	✓	✘
25	Finkel et al. (17)	✘	✓	✓	✓	NR
26	Fischer et al. (63)	✘	✓	✓	✓	✘
27	Franke et al. (17)	✘	✓	✓	✓	✘
28	Gilmore et al. (64)	✘	✓	✓	✓	✘
29	Gilmore et al. (65)	✘	✓	✓	✓	✘
30	Goodrich et al. (66)	✘	✓	✓	✓	✓
31	Han et al. (67)	✓	✓	✓	✓	✓
32	Hayes et al. (68)	✓	✓	✓	✓	NR
33	Heinzelmann et al. (69)	✘	✓	✓	✓	✘
34	Heltemes et al. (70)	✘	✓	✓	✓	✓
35	Hetherington et al. (71)	✘	✓	✓	✓	NR
36	Hoffer et al. (72)	✘	✓	✓	✓	NR
37	Huang et al. (73)	✘	✓	✓	✓	✓
38	Huang et al. (74)	✘	✓	✓	✓	✘
39	Janak et al. (75)	✓	✓	✓	✓	✓
40	Karch et al. (76)	✘	✓	✓	✓	✓
41	Kennedy et al. (77)	✘	✘	✓	✓	✓
42	Kennedy et al. (78)	✓	✓	✓	✓	✓
43	Kennedy et al. (79)	✓	✓	✓	✓	✓
44	Kontos et al. (80)	✘	✓	✓	✓	NR
45	Kontos et al. (81)	✘	✓	✓	✓	✓
46	Kontos et al. (82)	✘	✓	✓	✓	NR
47	Lange et al. (83)	✓	✓	✓	✓	✘
48	Lemke et al. (84)	✘	✘	✓	✓	✓
49	Levin et al. (85)	✘	✓	✓	✓	✘
50	Lew et al. (86)	✘	✓	✓	✓	NR
51	Lew et al. (87)	✘	✘	✓	✓	✘
52	Lew et al. (88)	✘	✓	✓	✓	NR
53	Licona et al. (89)	✘	✓	✓	✓	✓
54	Lindquist et al. (90)	✘	✓	✓	✓	NR
55	Lippa et al. (91)	✘	✓	✓	✓	✘
56	Luethcke et al. (92)	✘	✓	✓	✓	✓
57	MacDonald et al. (93)	✓	✓	✓	✓	✓
58	MacDonald et al. (94)	✘	✓	✓	✓	✘
59	MacDonald et al. (95)	✓	✓	✓	✓	✓
60	MacDonald et al. (96)	✓	✓	✓	✓	✓
61	MacDonald et al. (97)	✓	✓	✓	✓	✓
62	MacDonald et al. (98)	✘	✓	✓	✓	✘
63	Macera et al. (99)	✘	✓	✓	✓	NR
64	MacGregor et al. (100)	✘	✓	✓	✓	✓
65	MacGregor et al. (101)	✘	✘	✓	✓	✓
66	Magone et al. (102)	✓	✓	✓	✓	✓
67	Maguen et al. (103)	✓	✓	✓	✓	✘
68	Matthews et al. (104)	✘	✓	✓	✓	✘
69	Mendez et al. (105)	✘	✓	✓	✓	✘
70	Mendez et al. (106)	✘	✓	✓	✓	✘
71	Miller et al. (107)	✘	✓	✓	✓	✘
72	Morey et al. (108)	✘	✓	✓	✓	✘
73	Nathan et al. (109)	✘	✓	✓	✓	✘
74	Neipert et al. (110)	✘	✓	✓	✓	✘
75	Newsome et al. (111)	✘	✓	✓	✓	✘
76	Norris et al. (112)	✘	✘	✓	✓	✓
77	Norris et al. (113)	✘	✘	✓	✓	✓
78	Oleksiak et al. (114)	✘	✓	✓	✓	NR
79	O'Neil et al. (115)	✘	✓	✓	✓	✘
80	Petrie et al. (116)	✘	✓	✓	✓	✘
81	Pogoda et al. (117)	✘	✓	✓	✓	✓
82	Reid et al. (118)	✘	✓	✓	✓	✘
83	Riedy et al. (119)	✓	✓	✓	✓	✘
84	Robinson et al. (120)	✘	✓	✓	✓	✘
85	Ruff et al. (121)	✘	✓	✓	✓	NR
86	Ruff et al. (122)	✘	✘	✓	✓	✘
87	Ryu et al. (123)	✘	✓	✓	✓	✓
88	Saxe et al. (124)	✘	✓	✓	✓	✓
89	Scheibel et al. (125)	✘	✓	✓	✓	NR
90	Storzbach et al. (126)	✓	✓	✓	✓	✘
91	Stout et al. (127)	✘	✓	✓	✓	✘
92	Strigo et al. (128)	✘	✓	✓	✓	✘
93	Tate et al. (129)	✘	✓	✓	✓	✓
94	Trotter et al. (130)	✘	✓	✓	✓	NR
95	Troyanskaya et al. (131)	✘	✓	✓	✓	✘
96	Trudeau et al. (132)	✘	✓	✓	✓	NR
97	Vakhtin et al. (133)	✘	✓	✓	✓	NR
98	Verfaellie et al. (134)	✘	✓	✓	✓	✘
99	Verfaellie et al. (135)	✘	✓	✓	✓	✘
100	Walsh et al. (136)	✘	✓	✓	✓	✓
101	Wares et al. (137)	✘	✓	✓	✓	NR
102	Wilk et al. (138)	✘	✓	✓	✓	✓
103	Wilkinson et al. (139)	✘	✓	✓	✓	✘
104	Xydakis et al. (140)	✘	✓	✓	✓	✓
105	Yeh et al. (141)	✘	✓	✓	✓	✓
106	Yeh et al. (142)	✘	✓	✓	✓	✘

*✓Denotes the element was found within the study report. ✘ Denotes that the element was found to be within the study report, but did not meet the criteria (e.g., not complete). NR denotes unknown, unclear, or unreported. This means that the data element was not reported. NR does not necessarily indicate whether the element was part of the study, but inadvertently excluded from the report*.

Completeness indicates the presence or absence of key data elements from report i.e., sample size at the start of the study (excluded for records-based research); study time period; time since injury (i.e., time between injury and data points); control group; age of the study population (or age only reported as greater or less than with no mean, median, or range); and/or sex of the study population. This means that the data element was not reported but does not necessarily indicate whether the element was part of the study but inadvertently excluded from the report. Elements that were not considered key did not influence the indicator of completeness (e.g., branch of service, region TBI sustained, and GCS [since DoD no longer requires GCS for diagnosis]).

Correctness indicates whether there was a control or comparative group reported. If absent, this calls into question the quality of the data. However, although control or comparative groups were not reported in several studies, the data was in concordance with other high-quality studies (see concordance).

Concordance indicates the consistency of data reported as compared to a gold standard reference when taking currency into account [i.e., structured quantitative data and unstructured qualitative data related to outcomes were compared to those reported in the Veterans Health Administration, 2016 QUERI study; (143)]. Note that imaging outcomes were not reported in the VHA, 2016 QUERI study so imaging outcomes were not used to determine concordance.

Plausibility indicates whether data reported are within logical values. Logical values were considered as realistic values within possible limits for the methods cited within the study.

Currency indicates the minimum (median or average if minimum was not reported) time from injury (after rounding time reported for the study population which was not necessarily limited to blast-related mTBI) to data collection (if available). Times were reported in months. An average month was considered to be 4 weeks/30 days for conversion purposes where studies reported time in units besides months. Data were rounded up to nearest whole number if decimal over 0.5. NR indicates the data elements were unknown, unclear, or unreported. For records-based research the health records were assumed to be current unless otherwise reported (i.e., it is assumed that health records are reported at the time of or soon after the data are collected from the patient). For original research without use of health records, time since injury to data collection of over 3 months was considered as not current considering there is little evidence to suggest impairment beyond 3 months after a single mTBI (144–146). On occasion some report symptoms out to ~12 months (147). This is not meant to say that blast-related or non-blast-related mTBI cannot cause long-term outcomes, but simply cites that the time between sustaining the TBI and the minimum time to data collection was >3 months.

As shown in Table 3, 16% (*n* = 17) of the included studies were judged to be complete, 90% (*n* = 95) were judged to be correct, 100% were judged to be concordant, 100% were judged to be plausible, and 34% (*n* = 36) were judged to have currency. Across the five items, 8% (*n* = 9) of the included studies met all five items, 27% (*n* = 29) met four items, 59% (*n* = 63) met three items, and 5% (*n* = 5) met two items. No studies were judged to have met <1 item. Taken collectively, these figures suggest that the included studies matched with the goals of the current study.

### Data Synthesis and Statistical Analysis

Results were reported numerically and narratively to include a descriptive synthesis of the findings from the included studies. To facilitate appropriate comparisons, studies were first grouped according to whether they reported country-level data (derived from national hospital or registries) or field data. Other stratifications—for example, by study design and retrospective/prospective data collection–were also considered.

Findings are described overall, and broken down according to year of publication, military service, diagnostic criteria, age, and sex. Time trends of prevalence and outcomes of blast-related mTBI also are described. Data are presented in tables and figures. Meta-analysis was not possible owing to the heterogeneity of the studies.

## Results

### Description of Studies

The search strategy identified 1,860 unique records, of which 296 full text articles were assessed for eligibility. One hundred six of them met the criteria for inclusion in the review (Figure 1, PRISMA flowchart). Characteristics of the included studies and demonstration of data quality and epidemiological patterns of blast-related mTBI are summarized in Tables 3–8. The majority of studies included were published in 2007 or later with one exception published in 1998. Only 3 studies reported data on inactive Veterans from prior wars, the remaining focused primarily on TBIs sustained in Iraq and Afghanistan. Control or comparative groups were present in over 93% (*n* = 99) of the studies and consisted of healthy participants, with no TBI, with TBIs of varying severity, and/or caused by other mechanisms, and/or with other comorbidities.

All the studies included in this review were from U.S. investigators and utilized various inclusion criteria, case ascertainment sources, and TBI diagnostic and assessment modalities, which precluded comparative analyses. Of the 106 included studies, over 36% (38 studies) were conducted at, or leveraged data from specialty care clinics where brain imaging (e.g., MRI) was utilized to measure the presence and/or effects of blast-related mTBI at military or other medical facilities using self-report measures to analyze outcomes following injury and/or cognitive testing by a physician (Table 4). The remaining studies used data collected by physicians at the time of injury, by physicians or other specialists seen at specialty care clinics with a focus on treating patients with these types of injuries, by researchers, or by self-reported measures. All but three of the included studies were based out of Military or Veteran Facilities (Table 4).

**Table 4:** Characteristics of included studies.

**Study ID**	**Timeframe**	**Case ascertainment**			**Population at start**	**Blast-related mTBI**		**Assessment modality**				**Study setting category**
		**Medical records**	**Other assessment**	**Both**		***n***	**%**	**Self-report**	**Clinical assessment**	**Imaging**	**Medical records**	
Adam et al. (41)	3/2012–9/2012		✓		230	95	41%	✓	✓	✓		Military
Akin and Murnane (42)	NR		✓		NR	9		✓	✓			Civilian
Barlow-Ogden et al. (43)	NR		✓		NR	15			✓		✓	VA
Bazarian et al. (44)	8/2008–1/2010		✓		500	31	6%	✓		✓		VA
Belanger et al. (45)	NR			✓	137	38	28%	✓	✓		✓	VA
Belanger et al. (46)	NR			✓	640	298	47%	✓				Combination
Bell et al. (47)	4/2003–4/2008	✓			513	229	45%		✓			Military
Bjork et al. (48)	NR		✓		133	98	74%	✓	✓			VA
Bolzenius et al. (49)	NR			✓	40	12	30%	✓	✓	✓	✓	Civilian
Verfaellie et al. (50)	NR		✓		136	88	65%	✓	✓			Combination
Brenner et al. (51)	NR		✓		49	45	92%	✓	✓			Military
Callahan et al. (52)	NR			✓	87	42	48%	✓			✓	VA
Capo-Aponte et al. (53)	NR	✓			NR	20			✓			Military
Capo-Aponte et al. (54)	1/2008–2/2011	✓			549	343	62%	✓			✓	Military
Chen et al. (55)	10/2007–9/2009	✓			235	13	6%				✓	VA
Connelly et al. (24)	5/2006–7/2011	✓			43,852	1,950	4%		✓		✓	Military
Cooper et al. (56)	1/2008–1/2010	✓			120	32	27%	✓	✓		✓	Military
Davenport et al. (57)	3/2006–7/2007			✓	522	64	12%	✓		✓		Combination
Davenport et al. (57)	NR			✓	522	64	12%	✓		✓		Combination
Davenport et al. (**?** )2016)	NR			✓	124	54	44%	✓		✓	✓	Military
de Lanerolle et al. (59)	NR		✓		NR	25			✓	✓		Combination
Dretsch et al. (60)	2009		✓		71	34	48%	✓	✓			Military
Erickson (61)	11/2007–10/2009	✓			170	77	45%		✓			Military
Farrell-Carnahan et al. (62)	2008–2012			✓	424	114	27%	✓			✓	VA
Finkel et al. (17)	8/2008–12/2009	✓			NR	51			✓			Military
Fischer et al. (63)	NR		✓		107	21	20%	✓	✓	✓		VA
Franke et al. (17)	NR		✓		196	66	34%	✓	✓			Combination
Gilmore et al. (64)	NR			✓	127	127	100%	✓		✓	✓	Combination
Gilmore et al. (65)	NR		✓		124	60	48%	✓	✓			Combination
Goodrich et al. (66)	NR	✓			100	50	50%				✓	VA
Han et al. (67)	2008–2009 and 2010–2011		✓		124	103	83%			✓		Combination
Hayes et al. (68)	NR			✓	NR	59		✓	✓	✓		VA
Heinzelmann et al. (69)	NR	✓			117	19	16%	✓	✓		✓	Military
Heltemes et al. (70)	3/2004–3/2008	✓			1,129	473	42%				✓	Military
Hetherington et al. (71)	NR		✓		45	25	56%			✓		VA
Hoffer et al. (72)	NR		✓		127	60	47%	✓	✓			Military
Huang et al. (73)	NR			✓	99	23	23%	✓	✓	✓	✓	Military
Huang et al. (74)	NR		✓		NR	26			✓	✓		Combination
Janak et al. (75)	2008–2013	✓			2,502	159	6%	✓			✓	Military
Karch et al. (76)	1/2008–2/2011	✓			500	303	61%				✓	Military
Kennedy et al. (77)	1/2007–4/2009			✓	NR	274		✓			✓	Military
Kennedy et al. (78)	5/23/2005–8/31/2009	✓			926	586	63%				✓	Military
Kennedy et al. (79)	5/2010–11/2010		✓		377	342	91%		✓			Military
Kontos et al. (80)	11/2009–12/2011	✓			27,169	1,113	4%		✓			Military
Kontos et al. (81)	12/2009–3/2012		✓		276	19	7%		✓			Military
Kontos et al. (82)	NR			✓	50	23	46%		✓	✓		VA
Lange et al. (83)	2/2002–1/2009	✓			662	35	5%		✓			Military
Lemke et al. (84)	12/2006–1/2012	✓			64	60	94%		✓			VA
Levin et al. (85)	NR		✓		236	37	16%	✓		✓		VA
Lew et al. (86)	12/1999–7/2006	✓			NR	42			✓		✓	VA
Lew et al. (87)	12/2004–4/2008	✓			175	62	35%	✓	✓			VA
Lew et al. (88)	10/2007–6/2009	✓			36,919	10,431	28%	✓				VA
Licona et al. (89)	2005–2012			✓	8,293	84	1%	✓			✓	VA
Lindquist et al. (90)	2000–2011		✓		2,937	63	2%	✓				VA
Lippa et al. (91)	NR			✓	529	138	26%	✓			✓	VA
Luethcke et al. (92)	NR			✓	104	40	38%	✓	✓			Military
MacDonald et al. (93)	11/2008–10/2009			✓	122	63	52%			✓		Combination
MacDonald et al. (94)	NR			✓	NR	4		✓	✓	✓		Combination
MacDonald et al. (95)	2010–2013	✓			255	53	21%	✓	✓	✓		Combination
MacDonald et al. (96)	2008–2009			✓	122	63	52%	✓	✓		✓	Combination
MacDonald et al. (97)	3/2012–9/2012			✓	NR	38		✓	✓			Combination
MacDonald et al. (98)	2008–2013		✓		94	46	49%			✓		Combination
Macera et al. (99)	2008–2009	✓			5,5047	1,117	2%	✓				Military
MacGregor et al. (100)	1/2004–4/2008	✓			NR	1,822					✓	Military
MacGregor et al. (101)	2004–2008	✓			14,653	107	1%				✓	Military
Magone et al. (102)	1/1/2009–12/31/2011	✓			192	31	16%		✓			VA
Maguen et al. (103)	4/2007–1/2010	✓			1,713	390	23%	✓				VA
Matthews et al. (104)	NR		✓		NR	22		✓	✓	✓		VA
Mendez et al. (105)	NR		✓		NR	12		✓	✓	✓		VA
Mendez et al. (106)	NR		✓		150	12	8%	✓				VA
Miller et al. (107)	NR		✓		NR	53		✓		✓		Combination
Morey et al. (108)	10/12/2007–5/10/2010		✓		NR	11		✓		✓		Combination
Nathan et al. (109)	NR			✓	NR	186		✓		✓	✓	Military
Neipert et al. (110)	NR		✓		115	20	17%		✓			VA
Newsome et al. (111)	NR		✓		100	25	25%	✓	✓	✓		Combination
Norris et al. (112)	8/2010–3/2012	✓			990	210	21%	✓	✓			Military
Norris et al. (113)	NR	✓			NR	239		✓	✓			Military
Oleksiak et al. (114)	6/2007–7/2009	✓			250	154	62%		✓		✓	VA
O'Neil et al. (115)	9/2008–4/2011		✓		NR	47		✓				VA
Petrie et al. (116)	NR			✓	NR	34		✓	✓	✓		VA
Pogoda et al. (117)	10/1/2007–6/2009	✓			36,214	11,065	31%	✓	✓			VA
Reid et al. (118)	NR	✓			3,205	505	16%	✓			✓	Military
Riedy et al. (119)	8/1/2009–8/30/2014		✓		1,028	688	67%			✓	✓	Military
Robinson et al. (120)	NR		✓		203	20	10%	✓		✓		VA
Ruff et al. (121)	NR		✓		155	126	81%	✓	✓			VA
Ruff et al. (122))	NR		✓		126	74	59%	✓	✓			VA
Ryu et al. (123)	NR		✓		29	6	21%		✓			Combination
Saxe et al. (124)	10/2006–1/2008	✓			477	76	16%	✓			✓	Military
Scheibel et al. (125)	NR		✓		30	15	50%	✓	✓	✓		VA
Storzbach et al. (126)	9/2008–4/2011			✓	132	49	37%	✓	✓		✓	VA
Stout et al. (127)	NR		✓		57	20	35%			✓		Civilian
Strigo et al. (128)	NR		✓		36	18	50%	✓	✓	✓		VA
Tate et al. (129)	NR		✓		23	12	52%	✓		✓		Military
Trotter et al. (130)	NR		✓		350	45	13%	✓		✓		VA
Troyanskaya et al. (131)	NR		✓		97	54	56%		✓			VA
Trudeau et al. (132)	NR		✓		43	27	623%	✓	✓			VA
Vakhtin et al. (133)	NR		✓		67	13	19%	✓	✓	✓		VA
Verfaellie et al. (134)	NR		✓		95	67	71%	✓	✓			VA
Verfaellie et al. (135)	NR		✓		160	105	66%	✓	✓			VA
Walsh et al. (136)	1/2008–2/2011	✓			166	117	70%		✓			Military
Wares et al. (137)	NR		✓		169	86	51%	✓	✓			VA
Wilk et al. (138)	2006–2007		✓		7,668	424	6%	✓				Military
Wilkinson et al. (139)	NR	✓			92	26	28%	✓	✓			VA
Xydakis et al. (140)	NR		✓		1,555	136	9%		✓	✓		Military
Yeh et al. (141)	5/2009–6/2011			✓	NR	16		✓		✓		Military
Yeh et al. (142)	NR			✓	269	202	75%		✓	✓		Military

Table 5 presents the number of publications by year and the design of the studies. There are two noteworthy aspects of Table 5. First, among the included studies, 78% (*n* = 83) were published between 2009 and 2015. Within those 6 years, 46% (*n* = 38) were published in 2014 and 2015. Trends in the publication years of the included studies can be traced to two sources: (1) successive increases in VA-sponsored funding for TBI research following the ninth State-of-the-Art (SOTA) conference in 2008, which focused on advances in TBI science and (2) increased efforts to promote TBI research across scientific funding agencies [e.g., the 2014 National Research Action Plan (NRAP); (148)]. Second, despite increased in publication rates between 2009 and 2015, relative minor variability was observed in the research designs among the published studies. Across the 12-year range of the included studies, the most frequently used study design was prospective cohort (*n* = 62, 58%), followed by retrospective cohort (*n* = 37, 35%), case control (*n* = 4, 4%), and cross-sectional (*n* = 3, 3%).

**Table 5:** Number of included publications by year and study design.

**Publication year**	**#Pubs**	**Prospective cohort**	**Retrospective cohort**	**Case- control**	**Cross- sectional**
1998	1	1			
2007	1		1		
2008	1	1			
2009	5	3	2		
2010	6	3	3		
2011	9	2	6		1
2012	13	5	8		
2013	12	7	3	1	1
2014	19	10	7	2	
2015	19	16	2	1	
2016	12	9	3		
2017	8	5	2		1
Total	106	62	37	4	3

The study populations consisted of young adults. The lowest reported mean age was 23 years (60) and the highest was 37 years (42). As expected, the reported proportion of males was greater than females, ranging from 75% in an article on imaging patterns in U.S. military service members (94) to 100% (entirely male blast-related mTBI cohorts) in a series of articles (*n* = 31) (49, 55, 56, 61, 69, 70, 73, 74, 77, 81–83, 93, 95, 99, 104–106, 113, 116, 118, 123, 125, 128, 129, 132, 133, 137, 139, 142).

### Data Quality

Correctness, completeness, and currency are fundamental factors that describe the core concepts of health record data quality: if data is truthful, available, and the most recent available data (149, 150).

The quality of reporting in the final set of studies was found to be mixed (Table 3). Table 7 summarizes the findings from this review related to the completeness data quality factor. A set of variables were generally well-reported across all studies [e.g., n blast-related mTBI, Population (N) at End Total, Study Setting, Region TBI sustained, age, and sex]. Conversely, other relevant information that was indicative of study rigor (e.g., Time Since blast-related mTBI, Age Range blast-related mTBI, Study Period, and a clearly specified research design) were inconsistently reported in older papers. Reported outcomes are summarized in Table 8.

### Analyses of the Reported Outcomes From the Included Studies

#### Diagnosis and Assessment

This review included varied sources for mTBI diagnosis and assessment (Table 6): roughly 59% (*n* = 63) used clinical assessments; 66% (*n* = 70) used self-report; 36% (*n* = 38) relied on diagnostic imaging; 29% (*n* = 31) relied on reviews of medical charts. The majority (*n* = 99) used a combination of diagnostic and assessment modalities, while the remaining (*n* = 7) used a single modality for their studies.

**Table 6:** Number and percentage of diagnostic and assessment modalities used in the included articles.

**Diagnostic modalities**	**#**	**%**
Other Assessment	47	44%
Medical Records	34	32%
Both	25	24%
**Assessment modalities**		
Self-report	70	66%
Clinical assessment	63	59%
Imaging	38	36%
Medical records	31	29%
**Diagnostic and assessment modalities summary**		
Single modalities	7	7%
Combination of modalities (non-standard)	99	93%

*The categories for assessment modalities are not mutually exclusive meaning that the total will not be 100% (e.g., self-report can be combined with clinical assessment). Also, multiple methods may fall within the same modality (e.g., there are multiple methods that are considered imaging)*.

#### Mechanism of Injury

The number of blast-related mTBI participants included in the selected studies compared to the starting population ranged from 1 to 100% (*n* = 84/8,293 and *n* = 107/14,653 vs. *n* = 127/127, respectively).

#### Research Design and Data Collection Methods

Among the included studies that used non-blast TBI as a comparator or had no group for comparison (*n* = 35), ~57% included participants with blast-related mTBI compared to 43% non-blast mechanism participants. Of the studies included in this review, 54% (*n* = 57) reported the time between injury and study participation/data collection using at least one parameter (e.g., mean, median, or range). The mean time between injury and study participation ranged from the day of injury up to a maximum of 174 months.

#### Service Branch

In this review, 55% (*n* = 58) of the included studies reported the branch of service for participants. Of all studies, only 26% (*n* = 28) included service members from all branches. In addition, 18% (*n* = 18) of the studies included service members from 2 to 3 branches or components (including one study that had participants from both the National Guard and Army), and 8% (*n* = 9) included service members from the Army only.

#### Demographics

The majority of studies (66%, *n* = 70) reported the age of participants in some capacity (e.g., mean, median, or range, Supplementary Table 1). As shown in Table 7, most of the included studies (64%, *n* = 68) reported the sex of the participants with blast-related mTBI. While the reported proportion of males with blast-related mTBI was consistently greater than females, the proportion ranged from 75% in an article on imaging patterns in U.S. military service members (94) to 100% (entirely male blast-related mTBI cohorts) in a series of articles (*n* = 31) (49, 55, 56, 61, 69, 70, 73, 74, 77, 81–83, 93, 95, 99, 104–106, 113, 116, 118, 123, 125, 128, 129, 132, 133, 137, 139, 142).

**Table 7:** Data completeness in the included studies.

**Data element**	**# NR**	**Percent NR**
GCS	96	91%
Median age blast-related mTBI	94	89%
Median time since blast-related mTBI	90	85%
Age range blast-related mTBI	84	79%
Time since blast-related mTBI Range	75	71%
SD time since blast-related mTBI	68	64%
Rank	66	62%
Mean time since blast-related mTBI	65	61%
Branch of service	58	55%
Study period	57	54%
SD age blast-related mTBI	48	45%
Mean age blast-related mTBI	43	41%
*n* Male/Female blast-related mTBI	38	36%
Population (*N*) at start total	21	20%
Region TBI sustained	7	7%
Study setting	0	0%
Population (*N*) at end total	0	0%
*n* blast-related mTBI	0	0%

**Table 8:** The number of and percentage of major outcomes reported.

**Outcome**	**#Articles**	**% articles**
		**reporting**
PTSD	64	60%
Sensory impairments	35	33%
Depression	32	30%
Cognition	27	25%
Headaches	17	16%
Sleep disturbances	15	14%
White matter abnormalities/Functional connectivity	13	12%
Anxiety	6	6%

*The categories for outcomes reported are not mutually exclusive meaning that the total will not be 100% (i.e., multiple outcomes were analyzed in most studies)*.

#### Neurocognitive and Sensory Impairments

Visual deficits were measured in five studies (66, 88, 102, 117, 136). These studies reported visual impairments in 40%−68% of participants with photosensitivity and decreased visual acuity being the most commonly reported complaints (66, 88, 102, 117). Walsh et al. (136) reported at least one visual field defect (scatter, hemianopia, quadrantanopia, altitudinal, central, and constricted) in 64% of eyes examined in participants with blast-related mTBI (136). Other visual impairments reported include oculomotor dysfunction, floaters, pain, diplopia, and difficulty reading (66, 88, 102, 117).

#### Headaches

Our analysis included four studies looking specifically at prevalence of headache and seven others that reported headaches as an ancillary outcome. Of these studies of headache prevalence, the majority (over 60%) of participants with mTBI reported headache.

#### Comorbidities

Increased reports of these comorbidities were recorded for mTBI associated with blast, loss of consciousness, and multiple exposure. Of those observed in this review, PTSD had the highest prevalence with 64 studies reporting the condition, sensory impairments were the second most commonly reported (*n* = 35), and other common blast-related mTBI comorbidities included: depression (*n* = 33), cognitive deficits (*n* = 27), headaches/migraines (*n* = 17), and sleep disturbances (*n* = 15).

## Discussion

The primary goal of this review was to characterize the epidemiological patterns of blast-related mTBI and assess clinical outcomes in active U.S. military service members and inactive Veterans. Importantly, rigorous evaluation and confirmation of both mTBI and mechanism of injury (i.e., blast-related) are vital to producing a valid research study and to implement effective management and intervention strategies. However, it was evident from our review that blast-related mTBI studies are plagued by different and inconsistent diagnostic and assessment approaches. This issue has been noted in prior reviews (151), which suggests that it is an ongoing concern for the DoD medical community. Among the studies included in this review, the majority reported using self-report measures and a combination of diagnostic and assessment techniques. It should be noted that, in some cases, use of participants medical history was not possible because the study was conducted outside of the DoD or VA healthcare system or because the records were incomplete.

This analysis included studies that focused on multiple mechanisms of injury as well as some that were solely cohorts of blast-related injured active U.S. military service members and inactive Veterans; isolation of those studies [excluding studies where the Population (N) at the Start were not reported] revealed an average of 13% for blast-related mechanism with mTBI. Recent studies support a higher report rate (80%) in military cohorts with moderate-severe penetrating TBI (152).

Moreover, there was substantial variability of the inclusion criteria among the study samples. For example, we found a remarkable heterogeneity in terms of the number of blast-related mTBI participants included in the selected studies compared to the starting population. This was primarily due to the outcome measures used which included severity of specific outcomes of blast-related mTBI and prevalence of specific outcomes in mild–severe TBI resulting from various mechanisms of injury. Likewise, the composition of the comparative groups in the studies were also variable. Despite the observed variability in inclusion criteria and group composition, the participants in the included studies tended to be fairly homogenous: recently-deployed active duty participants with PTSD (57), combat Veterans who served in Operation Iraqi/Enduring Freedom (OIF/OEF) (43), and other military service members experiencing blast-related mTBI (43, 112).

That slightly higher than half of the studies reported the time between injury and study participation/data collection is important because the time immediately after injury is when symptoms tend to be most prominent. For example, in cases of acute mTBI, cognitive difficulties tend to diminish 3 months after injury (153).

Discrepancies were also observed in the reporting of the service branches of the participants in the included studies. Reporting participants service branch is important, not only for research purposes, but for policy and decision-making as well. For example, it is used to guide decision making about budget allocation for medical resources, education of service members and providers, research, and other areas. That the Army was prominently represented in the included studies is congruent with current and historical data from the DVBIC website (dvbic.dcoe.mil) which have shown that, among the major armed service branches, reports of TBI were most prevalent among the Army. In the first quarter of fiscal year 2018, 82% of all TBI reports were traced to the Army. The increase in mTBIs reported in other services, as well as the increase in reports among the female military population, is evidence of the spreading concern associated with the broad spectrum of TBIs and mTBIs in particular. A breakdown of the incidence and prevalence across branches of service was not calculated due to the limitations in the reported data.

It should be noted that, although the majority of studies reported the age of participants, the effect of age on the outcomes after any TBI is controversial. Some evidence suggests that younger individuals exhibit better recovery from TBI in general as compared to older adults. A study by Marquez de la Plata et al. (154) found that 16 to 26 year-olds and 27 to 39 year-olds showed better and faster improvement in functional abilities (e.g., following commands, performing daily activities, and engaging in recreational activities) as compared to individuals over 40 years, suggesting that neuroplasticity plays a role in the recovery process. Other research studies report discrepant findings that young adults are disproportionately affected by blast-related mTBIs. A growing body of literature suggests strong evidence from experimental and human studies that there is an association of an early life blast-related mTBI with late-onset neurodegenerative conditions and neuropathologic findings as a result of dynamic and persistent pathobiological processes and microstructural changes in the brain triggered by the injury (155–160). This will be a critical avenue for future investigation since such work would enhance our understanding of the long-term effects of blast-related concussion and encourage better management, strategic health service plans, and more effective preventive and therapeutic interventions to improve the quality of life of those serving in military conflicts.

Despite that more than half of the studies reported the sex of participants with blast-related mTBI, our analysis revealed inconsistencies in the representation of male and female study participants to the extent that there were males were disproportionately represented as compared with females. These findings are in line with the demographics of the U.S. military population where females make up ~14% across all services (161). This finding is significant for several reasons: (1) It highlights the need for additional research on mTBI in female military service members (162). (2) Women serving in the U.S. Military tend to be more diverse in their demographics, experiences, and health concerns as compared to women in the civilian population and men serving in the military (36). (3) Understanding sex-based differences in mTBI may have implications for recovery. For example, prior research has shown that, relative to male service members, female service members were more likely to report more post-concussion symptoms following mTBI (163–165) and were more likely to meet the criteria for post-concussional disorder (165, 166). Additional research suggests that, in some cases, females with mTBI are more likely than males with mTBI to report somatosensory and vestibular symptoms (167). Thus, the effects of blast-related mTBI on females is an important area for future investigation especially since changes in DoD policies will mean that more females will have been exposed to combat and therefore blast-related mTBI since 2015 (168).

Blast-related mTBI may be associated with impairments in sensory and neurocognitive functions. Studies included in this review focused on the prevalence of comorbidities and/or heightened deficits in one or more of the participants' faculties. The main comorbidities reported in the included studies were perceptual, neurological, and psychological. This finding is consistent with outcomes reported in prior reviews (151). Studies in this review that focused on the prevalence of comorbidities and/or heightened deficits reported high rates of multiple comorbid conditions including auditory and visual impairments, headaches, mental health conditions, and white matter changes and abnormalities. Taken collectively, the findings of this review agree with outcomes from prior research that found associations between blast-related mTBI and impairments in the sensory and cognitive faculties of military service members and Veterans.

Sensory impairments affect military service members readiness for deployment (169), job performance (170), and quality of life (171). The blast-related mTBI participants in these studies had a 100% report rate for some level of hearing disturbance immediately following the blast and symptoms persisted upwards of 6 months post-deployment (87, 88, 114, 117, 138). This finding is consistent with hearing loss being the most commonly compensated complaint among U.S. Veterans Administration beneficiaries (172). Although each study reported a need for further, more extensive research into blast-related mTBIs impact on vision, the consensus was, without considering other mitigating factors, visual impairments improve over time. Thus, although visual impairments may be a measure of blast-related mTBI recovery and could be useful in the rehabilitation process, blast mechanism does not seem to be predictive of visual difficulties. Some researchers have raised concerns about the lack of ICD-10 codes for specific visual and auditory deficits associated with TBI (173).

Another commonly reported outcome in the included studies was post-traumatic and/or comorbid headaches. As mentioned, four of the included studies focused specifically on headaches and three others reported headaches as an ancillary outcome. One study reported that, among a cohort of 96 active duty service members with mTBI, the most common type of headache was “continuous type with migraine features” (*n* = 31, 18.7%) and the least common was “headaches not otherwise classifiable” (*n* = 5, 3.0%) (93). Frequent headaches are associated with an increased need for medical care (174) and a diminished quality of life (175). However, it should be noted that there was no evidence that blast-related injuries increased the likelihood of headaches (151). Interestingly, sleep was found to have the biggest impact on reduction and recovery of the headaches (17, 121, 122, 174).

Among the included studies that leveraged imaging, the most commonly reported neurological abnormalities were white matter irregularities, cerebellar damage, thalamic network architectural differences, metabolic activation, diffuse axonal injury (DAI), and sensorimotor impairment (68, 93, 94, 107–109, 119, 120, 127, 133). Studies compared imaging outcomes from injured and non-injured participants on a range of abilities including: pain perception (123), cognition (41, 45), personality (57), and symptoms of PTSD (130). Outcomes from these studies can be used to develop better preventive devices and to develop targeted treatments.

Due to the nature and conditions in which the injury occurred (e.g., co-occurring battle wounds, conditions that are a product of injuries to the brain, and experiences surrounding the injury), comorbid conditions are common in military service members with blast-related mTBIs. Research is ongoing and more data is needed to distinguish the contributions of pure blast overpressure and blunt trauma in blast-related TBI. The most commonly reported comorbid and pre-existing conditions associated with blast-related mTBI were mental health conditions, including PTSD, depression, anxiety, sleep disorders, attention disorders, and cognitive disorders (44, 55, 62, 65, 77, 78, 92, 103, 115, 124, 134, 135, 176, 177).

U.S. Veterans Administration beneficiaries with mTBI are likely to have comorbid conditions which can drive up the cost of care (178). To explore and provide recommendations that address blast-related mTBI among U.S. Veterans Administration beneficiaries and services members is beyond the scope of this study; however, recommendations by others included providing greater opportunities for rehabilitation and training services to help injured service members transition to a productive civilian life (179). Future studies could provide additional insight into this topic. Of particular note, future efforts should aim to consistently report the Veteran status of participants. The term “Veteran” was inconsistently used across studies included in this review thus limiting the comparison of outcomes among active U.S. military service member and inactive Veterans.

While the bulk of studies selected for this review focused on diagnostics, treatment, and technology, few addressed the significant financial impact of brain injuries resulting from blast exposures. A recent study by Dismuke-Greer et al. (180) found that outpatient treatment costs for veterans with blast-related mTBI was significantly higher ($6,480; 95% CI, $5,842–$7,187) than outpatient treatment costs for veterans with non-blast-related mTBI ($4,901; 95% CI, $4,392–$5,468) (180). From an economic perspective, the cost of treating all TBI severities is significant. While estimates vary greatly and are severely limited, the 2008 estimates (using 2007 U.S. dollars) revealed costs per case for 1 year following TBI to be ranging between $27,259 and $32,759 for mTBI and up to $408,519 for moderate and severe TBI with the acute hospital care costs ranging from $15,144 to $21,246 for mTBI (181). These cost estimates do not factor in the cost of prevention, protection, pre- and post-deployment screening, or other current and emerging costs associated with mental health conditions. As mentioned previously, TBI in general is often accompanied by other comorbidities such as PTSD that itself is costly to treat. For example, in 2008 the RAND Corporation estimated that baseline cost to treat PTSD alone for 50,000 E-5s (e.g., Army Sergeant) was $204,691,652 (including lives lost to suicide) and $119,829,381 (excluding lives lost to suicide) (181). In the same report, they estimated that the total cost to treat deployment-related TBI was between $554–$854 million dollars and that 47–57% of the total cost is due to productivity loss (181).

In addition to clinical treatment cost and productivity loss, consideration must be given to the cost to fund research studies to further knowledge of TBI. For example, in 2018 the U.S. government appropriated $125 million to support the Department of Defense Psychological Health/Traumatic Brain Injury Research Program (CDMRP). The important point is that personal and societal cost of treating all forms of TBI is significant. For comparison, the average cost per year per case of multiple sclerosis is estimated at $69,000 (Society^1^) including productivity loss whereas the cost of cardiovascular disease per year per case is estimated at $18,953 (182, 183). Using data from 16 European Union states, researchers calculated 374,636 years of life lost due to TBI-related deaths (184).

### Limitations

Confounding the limitations inherent in the studies themselves, this review was limited in that raw data were not available from all included studies. Studies reviewed did not conform to one approach for confirming participant's TBI diagnoses. More specifically, the documentation of mechanisms was primarily identified through self-report methods. Therefore, this analysis may include misdiagnosed and miscategorized instances of blast-related mTBI, as reported in the included studies, as well as underreporting of mTBI. The exclusion of portions of demographic data in the studies reviewed to include branch of service could have created bias. With limited data available to characterize the populations from all studies there is a possibility for overlapping populations to be included in this review. Additional studies are warranted to examine the military occupational specialty (MOS) to allow an analysis of the rates of blast-related mTBI among sub-populations with blast-related mTBI. Accumulative effects should also be addressed in future studies.

Since 2006 there have been several changes to DoD policies related to the broad spectrum of TBI to include changes in the DoD definition of mTBI. Resulting education and training on the new definition may have impacted the reported diagnoses in studies of individuals diagnosed before and after the definition changed.

The most important limiting factors hampering research and clinical care of blast-related mTBI are determining physiological from pathological changes: lack of a dose response curve defining exposure to primary blast to injury (185), and the current inability to distinguish the contributions of the secondary to quinary effects from the primary blast, in the human.

Our systematic review focused on U.S. military service members who experienced blast-related mTBI. Unlike other forms of TBI injury (186, 187), no shift or epidemiological transition of this population has been witnessed over the years, making these participants distinct from civilians affected by TBI. While it should be noted that studies have investigated blast-related injuries in civilian samples (188, 189), the fact that the studies under review are limited to primarily military samples may limit the generalizability and clinical applicability of the findings for civilian populations.

### Outlook: Implications for Practice and Research

One implication of the findings of this study pertaining to health care practice and research is related to the standardization of diagnostics and reporting of those diagnoses. First, poor or a lack of documentation (e.g., diagnosis and TBI disability severity rating) impedes service members ability to receive care (190). Second, while all studies entailed some sort of clinical assessment, imaging, records, and/or formal interviews with medical specialists as the major diagnostic means, the tools leveraged to accomplish the diagnoses were highly variable from study-to-study which limited comparison across studies. Applying minimal data management standards for epidemiological studies of blast injury consistent with Bieler et al. would address this issue and facilitate use of a broader range of datasets for longitudinal studies (7).

A related implication of the current study is the need for greater specificity in the diagnostic process. Details about the injuring event, including the proximity of the injured person to the blast and the amount of force experienced (151), could facilitate not only a more precise definition of “blast exposure,” but clinical and medical personnel's ability to assess the extent of the injury. One way to accomplish this would be to focus research and development efforts on outfitting service members' helmets with more robust sensors that are capable of recording force (191). At the same time, there is also a need for greater understanding of outcomes resulting from acute, chronic, and cumulative blast exposures.

Another implication of the findings of this study, particularly with regard to the prevalence findings, is that health care workers should be informed about the issues and challenges that are unique to military service members so that they can provide appropriate treatment (192). This study also reinforces that service members should be using up-to-date protective headgear because it may reduce mortality and severity of TBI (193). It is interesting to note that, even as the state of art of defense weaponry evolves, there remain significant risks to both the target and operator (194). For example, so-called “non-lethal” weapons (e.g., acoustic weapons, laser weapons), can result in TBI-related injuries (195). Thus, it is imperative that service members are equipped to deal with the advancing state of military weaponry and the shifting conditions in which they are encountered and follow the prescribe usage of both weapon systems and protective/preventative equipment.

Furthermore, the military or combat setting is not the only setting for blast-related mTBI. The importance of these findings is increasingly important to civilians because of the frequency of blast-related accidents and incidents as well as use of explosive devices in terrorist attacks.

## Author Contributions

HP and SM developed and executed the protocol and search strategy, completed the title and abstract reviews as well as extracted, and the full texts. HA served as a third reviewer in the case of discrepancies during title, abstract, and full text reviews. AW, CM, JH, KT, MC, AH, and HP extracted data from included articles and assisted in drafting the narrative. NR, JN, and PS validated the extracted data and revised the narrative. PS built the tables. HP, SM, TD, FK, and SH revised the final narrative. All authors contributed to the article and approved the submitted version.

## Conflict of Interest

HP, AW, TD, NR, PS, JN, CM, JH, KT, and MC were employed by Booz Allen Hamilton. AH was employed by Dell. The remaining authors declare that the research was conducted in the absence of any commercial or financial relationships that could be construed as a potential conflict of interest.

## Footnotes

^1^*Financial Burdens for People with MS, their Families, and Society*.

## References

[1] Jones E Fear NT Wessely S. Shell shock and mild traumatic brain injury: a historical review. Am J Psychiatry. (2007) 164:1641–5. 10.1176/appi.ajp.2007.07071180 17974926

[2] Brundage JF Taubman SB Hunt DJ Clark LL. (2015) Whither the “signature wounds of the war” after the war: estimates of incidence rates and proportions of TBI and PTSD diagnoses attributable to background risk, enhanced ascertainment, and active war zone service, active component, armed forces US. 2003–2014. MSMR. 22:2–11. 25734618

[3] Office DBIRC FY18 Prevention, Mitigation, and Treatment of Blast Injuries Report to the Executive Agent ed. Amedd (2019).

[4] Office DBIRC What is Blast Injury? Ft Detrick: US Army Medical Research and Development Command (2019). Available online at: https://blastinjuryresearch.amedd.army.mil/index.cfm/blast_injury_101/what_is_blast_injury (accessed January 13, 2020).

[5] DoD DoD Doctrine 6025.21E, Medical Research for Prevention, Mitigation, and Treatment of Blast Injuries (2006).

[6] Dennis AM Kochanek PM Pathobiology of Blast Injury. New York, NY: Springer (2007). 10.1007/978-3-540-49433-1_92

[7] Bieler D Cernak I Martineau L Bjarnason S Franke A Kirkman E. Guidelines for conducting epidemiological studies of blast injury. J R Army Med Corps. (2019) 165:41–44. 10.1136/jramc-2018-000948 29666201

[8] Hoge CW Mcgurk D Thomas JL Cox AL Engel CC Castro CA. Mild traumatic brain injury in U.S. soldiers returning from iraq. N Engl J Med. (2008) 358:453–63. 10.1056/NEJMoa072972 18234750

[9] Fralick M Sy E Hassan A Burke MJ Mostofsky E Karsies T. Association of concussion with the risk of suicide: a systematic review and meta-analysis. JAMA Neurol. (2019) 76:144–51. 10.1001/jamaneurol.2018.3487 30419085 PMC6439954

[10] Langlois JA Rutland-Brown W Wald MM. The epidemiology and impact of traumatic brain injury: a brief overview. J Head Trauma Rehabil. (2006) 21:375–8. 10.1097/00001199-200609000-00001 16983222

[11] CDC Severe TBI. (2017). Available online at: https://www.cdc.gov/traumaticbraininjury/severe.html (accessed May 10, 2018).

[12] Eibner C Ringel JS Kilmer B Pacula RL Diaz C The cost of post-deployment mental health and cognitive conditions. In: Jaycox TTLH editor. Invisible Wounds of War: Psychological and Cognitive Injuries, Their Consequences, and Services to Assist Recovery. Santa Monica, CA: Rand (2008). p 1–499.

[13] DVBIC. DoD Worldwide Numbers for TBI. Falls Church, VA (2020). Available online at: https://dvbic.dcoe.mil/dod-worldwide-numbers-tbi (accessed July 16 2020).

[14] Excellence DCF DCoE Clinical Recommendation, July 2013: Neuroimaging following Mild Traumatic Brain Injury in the Non-Deployed Setting (2013).

[15] Vanderploeg RD Belanger HG Horner RD Spehar AM Powell-Cope G Luther SL. Health outcomes associated with military deployment: mild traumatic brain injury, blast, trauma, and combat associations in the florida national guard. Arch Phys Med Rehabil. (2012) 93:1887–95. 10.1016/j.apmr.2012.05.024 22705240

[16] Walker WC Franke LM Mcdonald SD Sima AP Keyser-Marcus L. Prevalence of mental health conditions after military blast exposure, their co-occurrence, and their relation to mild traumatic brain injury. Brain Inj. (2015) 29:1581–8. 10.3109/02699052.2015.1075151 26479126

[17] Finkel AG Yerry JA Klaric JS Ivins BJ Scher A Choi YS. Headache in military service members with a history of mild traumatic brain injury: a cohort study of diagnosis and classification. Cephalalgia. (2017) 37:548–59. 10.1177/0333102416651285 27206963

[18] Swanson TM Isaacson BM Cyborski CM French LM Tsao JW Pasquina PF. Traumatic brain injury incidence, clinical overview, and policies in the US military health system since 2000. Public Health Rep. (2017) 132:251–9. 10.1177/0033354916687748 28135424 PMC5349478

[19] Vickers ML Coorey CP Milinovich GJ Eriksson L Assoum M Reade MC. Bibliometric analysis of military trauma publications: 2000-2016. J R Army Med Corps. (2018) 164:142–9. 10.1136/jramc-2017-000858 29331949

[20] Taylor BC Hagel EM Carlson KF Cifu DX Cutting A Bidelspach DE. Prevalence and costs of co-occurring traumatic brain injury with and without psychiatric disturbance and pain among afghanistan and iraq war veteran V.A. users. Med Care. (2012) 50:342–6. 10.1097/MLR.0b013e318245a558 22228249

[21] DVBIC. DoD Worldwide Numbers for TBI. Falls Church, VA (2020). Available online at: https://dvbic.dcoe.mil/dod-worldwide-numbers-tbi (accessed August 4, 2020).

[22] CDC N DoD and Va Leadership Panel Report to Congress on Traumatic Brain Injury in the United States: Understanding the Public Health Problem Among Current and Former Military Personnel (2013).

[23] Agimi Y Regasa LE Stout KC. Incidence of traumatic brain injury in the military US. 2010–2014. Mil Med. (2019) 184:e233–41. 10.1093/milmed/usy313 30517721

[24] Connelly C Martin K Elterman J Zonies D. Early traumatic brain injury screen in 6594 inpatient combat casualties. Injury. (2017) 48:64–69. 10.1016/j.injury.2016.08.025 27639602

[25] Peskind ER Brody D Cernak I Mckee A Ruff RL. Military- and sports-related mild traumatic brain injury: clinical presentation, management, long-term consequences. J Clin Psychiatry. (2013) 74:180–8. 10.4088/JCP.12011co1c 23473351 PMC5904388

[26] Scofield DE Proctor SP Kardouni JR Hill OT Mckinnon CJ. Risk factors for mild traumatic brain injury and subsequent post-traumatic stress disorder and mental health disorders among united states army soldiers. J Neurotrauma. (2017) 34:3249–55. 10.1089/neu.2017.5101 28895451

[27] Gray SN. An overview of the use of neurofeedback biofeedback for the treatment of symptoms of traumatic brain injury in military and civilian populations. Med Acupunct. (2017) 29:215–19. 10.1089/acu.2017.1220 28874922 PMC5580369

[28] Williams VF Stahlman S Hunt DJ O'donnell FL Diagnoses of traumatic brain injury not clearly associated with deployment, active component, armed forces US. 2001–2016. MSMR. (2017) 24:2–8. 28358519

[29] Boutte AM Thangavelu B Lavalle CR Nemes J Gilsdorf J Shear DA. Brain-related proteins as serum biomarkers of acute, subconcussive blast overpressure exposure: a cohort study of military personnel. PLoS ONE. (2019) 14:e0221036. 10.1371/journal.pone.0221036 31408492 PMC6692016

[30] Bricknell M. Fundamentals of military medicine: a new resource from the US army borden institute. BMJ Mil Health. (2020) 166:284. 10.1136/jramc-2019-001289 32015182

[31] Moher D Liberati A Tetzlaff J Altman DG Group P Preferred reporting items for systematic reviews and meta-analyses: the PRISMA statement. BMJ. (2009) 339:b2535 10.1136/bmj.b2535 19622551 PMC2714657

[32] Higgins J Green S Cochrane Handbook for Systematic Reviews of Interventions. Oxford: The Cochrane Collaboration (2011).

[33] DoD DoD Instruction 6490.11, DoD Policy Guidance for Management of Mild Traumatic Brain Injury/Concussion in the Deployed Setting (2012).

[34] DoD DoD Instruction 6490.13, Comprehensive Policy on Traumatic Brain Injury-Related Neurocognitive Assessments by the Military Services (2017).

[35] DoD/VA. VA/DoD Clinical Practice Guideline for Management of Concussion/mild Traumatic Brain Injury. Washington, DC: DoD/VA. (2009). 20108447

[36] Danan ER Krebs EE Ensrud K Krebs E Koeller E Greer N. An evidence map of the women veterans' health research literature (2008–2015). J Gen Intern Med. (2017) 32:1359–76. 10.1007/s11606-017-4152-5 28913683 PMC5698220

[37] Medicine TACOR Definition of mild traumatic brain injury. J Head Trauma Rehabil. (1993) 8:86–7. 10.1097/00001199-199309000-00010

[38] Holm L Cassidy JD Carroll LJ Borg J Neurotrauma Task Force on Mild Traumatic Brain Injury of The WHOCC. Summary of the WHO collaborating centre for neurotrauma task force on mild traumatic brain injury. J Rehabil Med. (2005) 37:137–41. 10.1080/16501970510027321 16040469

[39] DoD Medical Research for Prevention, Mitigation, and Treatment of Blast Injuries (2018).

[40] Weiskopf NG Hripcsak G Swaminathan S Weng C. Defining and measuring completeness of electronic health records for secondary use. J Biomed Inform. (2013) 46:830–6. 10.1016/j.jbi.2013.06.010 23820016 PMC3810243

[41] Adam O Mac Donald CL Rivet D Ritter J May T Barefield M. Clinical and imaging assessment of acute combat mild traumatic brain injury in afghanistan. Neurology. (2015) 85:219–27. 10.1212/WNL.0000000000001758 26109715 PMC4516289

[42] Akin FW Murnane OD. Head injury and blast exposure: vestibular consequences. Otolaryngol Clin North Am. (2011) 44:323–34. 10.1016/j.otc.2011.01.005 21474007

[43] Barlow-Ogden K Poynter W. Mild traumatic brain injury and posttraumatic stress disorder: investigation of visual attention in operation iraqi freedom/operation enduring freedom veterans. J Rehabil Res Dev. (2012) 49:1101–14. 10.1682/JRRD.2010.09.0188 23341282

[44] Bazarian JJ Donnelly K Peterson DR Warner GC Zhu T Zhong JH. The relation between posttraumatic stress disorder and mild traumatic brain injury acquired during operations enduring freedom and iraqi freedom. J. Head Trauma Rehabil. (2013) 28:1–12. 10.1097/HTR.0b013e318256d3d3 22647965

[45] Belanger HG Kretzmer T Yoash-Gantz R Pickett T Tupler LA. Cognitive sequelae of blast-related versus other mechanisms of brain trauma. J Int Neuropsychol Soc. (2009) 15:1–8. 10.1017/S1355617708090036 19128523

[46] Belanger HG Proctor-Weber Z Kretzmer T Kim M French LM Vanderploeg RD. Symptom complaints following reports of blast versus nonblast mild TBI: does mechanism of injury matter? Clin Neuropsychol. (2011) 25:702–15. 10.1080/13854046.2011.566892 21512958

[47] Bell RS Vo AH Neal CJ Tigno J Roberts R Mossop C. Military traumatic brain and spinal column injury: a 5-year study of the impact blast and other military grade weaponry on the central nervous system. J Trauma. (2009) 66(4 Suppl):S104–11. 10.1097/TA.0b013e31819d88c8 19359953

[48] Bjork JM Burroughs TK Franke LM Pickett TC Johns SE Moeller FG. Laboratory impulsivity and depression in blast-exposed military personnel with post-concussion syndrome. Psychiatry Res. (2016) 246:321–5. 10.1016/j.psychres.2016.10.008 27750113

[49] Bolzenius JD Roskos PT Salminen LE Paul RH Bucholz RD. Cognitive and self-reported psychological outcomes of blast-induced mild traumatic brain injury in veterans: a preliminary study. Appl Neuropsychol Adult. (2015) 22:79–87. 10.1080/23279095.2013.845823 24940794

[50] Verfaellie M Lafleche G Spiro A Bousquet K Neuropsychological outcomes in OEF/OIF veterans with self-report of blast exposure: associations with mental health, but not MTBI. Neuropsychology. (2014) 28:337–46. 10.1037/neu0000027 24245929

[51] Brenner LA Terrio H Homaifar BY Gutierrez PM Staves PJ Harwood JEF. Neuropsychological test performance in soldiers with blast-related mild TBI. Neuropsychology. (2010) 24:160–7. 10.1037/a0017966 20230110

[52] Callahan ML Binder LM O'Neil ME Zaccari B Roost MS Golshan S. Sensory sensitivity in operation enduring freedom/operation Iraqi freedom veterans with and without blast exposure and mild traumatic brain injury. Appl Neuropsychol Adult. (2016) 25:126–36. 10.1080/23279095.2016.1261867 27929660

[53] Capó-Aponte JE Urosevich TG Temme LA Tarbett AK Sanghera NK. Visual dysfunctions and symptoms during the subacute stage of blast-induced mild traumatic brain injury. Mil Med. (2012) 177:804–13. 10.7205/MILMED-D-12-00061 22808887

[54] Capó-Aponte JE Jorgensen-Wagers KL Sosa JA Walsh DV Goodrich GL Temme LA. Visual dysfunctions at different stages after blast and non-blast mild traumatic brain injury. Optom Vis Sci. (2017) 94:7–15. 10.1097/OPX.0000000000000825 26889821

[55] Chen LL Baca CB Choe J Chen JW Ayad ME Cheng EM. Posttraumatic epilepsy in operation enduring freedom/operation iraqi freedom veterans. Mil Med. (2014) 179:492–6. 10.7205/MILMED-D-13-00413 24806494

[56] Cooper DB Chau PM Armistead-Jehle P Vanderploeg RD Bowles AO. Relationship between mechanism of injury and neurocognitive functioning in OEF/OIF service members with mild traumatic brain injuries. Mil Med. (2012) 177:1157–60. 10.7205/MILMED-D-12-00098 23113441

[57] Davenport ND Lim KO Sponheim SR. Personality and neuroimaging measures differentiate PTSD from mTBI in veterans. Brain Imaging Behav. (2015) 9:472–83. 10.1007/s11682-015-9371-y 25796167

[58] Davenport ND Lamberty GJ Nelson NW Lim KO Armstrong MT Sponheim SR. PTSD confounds detection of compromised cerebral white matter integrity in military veterans reporting a history of mild traumatic brain injury. Brain Inj. (2016) 30:1491–500. 10.1080/02699052.2016.1219057 27834537

[59] de Lanerolle NC Hamid H Kulas J Pan JW Czlapinski R Rinaldi A. Concussive brain injury from explosive blast. Ann Clin Transl Neurol. (2014) 1:692–702. 10.1002/acn3.98 25493283 PMC4241796

[60] Dretsch MN Kelly MP Coldren RL Parish RV Russell ML. No significant acute and subacute differences between blast and blunt concussions across multiple neurocognitive measures and symptoms in deployed soldiers. J Neurotrauma. (2015) 32:1217–22. 10.1089/neu.2014.3637 25367048

[61] Erickson JC. Treatment outcomes of chronic post-traumatic headaches after mild head trauma in US soldiers: an observational study. Headache. (2011) 51:932–44. 10.1111/j.1526-4610.2011.01909.x 21592097

[62] Farrell-Carnahan L Franke L Graham C Mcnamee S. Subjective sleep disturbance in veterans receiving care in the veterans affairs polytrauma system following blast-related mild traumatic brain injury. Mil Med. (2013) 178:951–6. 10.7205/MILMED-D-13-00037 24005542

[63] Fischer BL Parsons M Durgerian S Reece C Mourany L Lowe MJ. Neural activation during response inhibition differentiates blast from mechanical causes of mild to moderate traumatic brain injury. J Neurotrauma. (2014) 31:169–79. 10.1089/neu.2013.2877 24020449 PMC3900006

[64] Gilmore CS Camchong J Davenport ND Nelson NW Kardon RH Lim KO. Deficits in visual system functional connectivity after blast-related mild tbi are associated with injury severity and executive dysfunction. Brain Behav. (2016) 6:e00454. 10.1002/brb3.454 27257516 PMC4873652

[65] Gilmore CS Marquardt CA Kang SS Sponheim SR. Reduced P3b brain response during sustained visual attention is associated with remote blast mTBI and current PTSD in U.S. military veterans. Behav Brain Res. (2016). 340:174–82. 10.1016/j.bbr.2016.12.002 27931783 PMC11778509

[66] Goodrich GL Flyg HM Kirby JE Chang CY Martinsen GL. Mechanisms of TBI and visual consequences in military and veteran populations. Optom Vis Sci. (2013) 90:105–12. 10.1097/OPX.0b013e31827f15a1 23314131

[67] Han K Mac Donald CL Johnson AM Barnes Y Wierzechowski L Zonies D. Disrupted modular organization of resting-state cortical functional connectivity in U.S. military personnel following concussive ‘mild' blastrelated traumatic brain injury. Neuroimage. (2014) 84:76–96. 10.1016/j.neuroimage.2013.08.017 23968735 PMC3849319

[68] Hayes JP Miller DR Lafleche G Salat DH Verfaellie M. The nature of white matter abnormalities in blast-related mild traumatic brain injury. Neuroimage. (2015) 8:148–56. 10.1016/j.nicl.2015.04.001 26106539 PMC4473287

[69] Heinzelmann M Reddy SY French LM Wang D Lee H Barr T. Military personnel with chronic symptoms following blast traumatic brain injury have differential expression of neuronal recovery and epidermal growth factor receptor genes. Front Neurol. (2014) 5:198. 10.3389/fneur.2014.00198 25346719 PMC4191187

[70] Heltemes KJ Holbrook TL MacGregor AJ Galarneau MR. Blast-related mild traumatic brain injury is associated with a decline in self-rated health amongst US military personnel. Injury. (2012) 43:1990–5. 10.1016/j.injury.2011.07.021 21855064

[71] Hetherington HP Hamid H Kulas J Ling G Bandak F de Lanerolle NC. MRSI of the medial temporal lobe at 7 T in explosive blast mild traumatic brain injury. Magn Reson Med. (2014) 71:1358–67. 10.1002/mrm.24814 23918077 PMC4117409

[72] Hoffer ME Donaldson C Gottshall KR Balaban C Balough BJ. Blunt and blast head trauma: different entities. Int Tinnitus J. (2009) 15:115–8. 20420334

[73] Huang MX Nichols S Robb A Angeles A Drake A Holland M. An automatic MEG low-frequency source imaging approach for detecting injuries in mild and moderate TBI patients with blast and non-blast causes. Neuroimage. (2012) 61:1067–82. 10.1016/j.neuroimage.2012.04.029 22542638

[74] Huang MX Harrington DL Robb Swan A Angeles Quinto A Nichols S Drake A. Resting-state magnetoencephalography reveals different patterns of aberrant functional connectivity in combat-related mild traumatic brain injury. J Neurotrauma. (2017) 34:1412–26. 10.1089/neu.2016.4581 27762653

[75] Janak JC Cooper DB Bowles AO Alamgir AH Cooper SP Gabriel KP. Completion of multidisciplinary treatment for persistent postconcussive symptoms is associated with reduced symptom burden. J Head Trauma Rehabil. (2015) 32:1–15. 10.1097/HTR.0000000000000202 26709579

[76] Karch SJ Capó-Aponte JE McIlwain DS Lo M Krishnamurti S Staton RN. Hearing loss and tinnitus in military personnel with deployment-related mild traumatic brain injury. US Army Med Dep J. (2016) 2016:52–63. 27613210

[77] Kennedy JE Cullen MA Amador RR Huey JC Leal FO. Symptoms in military service members after blast mTBI with and without associated injuries. NeuroRehabilitation. (2010) 26:191–7. 10.3233/NRE-2010-0555 20448309

[78] Kennedy JE Leal FO Lewis JD Cullen MA Amador RR. Posttraumatic stress symptoms in OIF/OEF service members with blast-related and non-blast-related mild TBI. NeuroRehabilitation. (2010) 26:223–31. 10.3233/NRE-2010-0558 20448312

[79] Kennedy CH Porter Evans J Chee S Moore JL Barth JT Stuessi KA. Return to combat duty after concussive blast injury. Arch Clin Neuropsychol. (2012) 27:817–27. 10.1093/arclin/acs092 23059351

[80] Kontos AP Kotwal RS Elbin RJ Lutz RH Forsten RD Benson RJ. Residual effects of combat-related mild traumatic brain injury. J Neurotrauma. (2013) 30:680–6. 10.1089/neu.2012.2506 23031200

[81] Kontos AP Elbin RJ Kotwal RS Lutz RH Kane S Benson PJ. The effects of combat-related mild traumatic brain injury (mTBI): does blast mTBI history matter? J Trauma Acute Care Surg. (2015) 79:S146–51. 10.1097/TA.0000000000000667 26131789

[82] Kontos AP Van Cott AC Roberts J Pan JW Kelly MB Mcallister-Deitrick J. Clinical and magnetic resonance spectroscopic imaging findings in veterans with blast mild traumatic brain injury and post-traumatic stress disorder. Mil Med. (2017) 182:99–104. 10.7205/MILMED-D-16-00177 28291459 PMC6946024

[83] Lange RT Pancholi S Brickell TA Sakura S Bhagwat A Merritt V. Neuropsychological outcome from blast versus non-blast: mild traumatic brain injury in U.S. military service members. J Int Neuropsychol Soc. (2012) 18:595–605. 10.1017/S1355617712000239 22459022

[84] Lemke S Cockerham GC Glynn-Milley C Cockerham KP. Visual quality of life in veterans with blast-induced traumatic brain injury. JAMA Ophthalmol. (2013) 131:1602–9. 10.1001/jamaophthalmol.2013.5028 24136237

[85] Levin HS Wilde E Troyanskaya M Petersen NJ Scheibel R Newsome M. Diffusion tensor imaging of mild to moderate blast-related traumatic brain injury and its sequelae. J Neurotrauma. (2010) 27:683–94. 10.1089/neu.2009.1073 20088647

[86] Lew HL Jerger JF Guillory SB Henry JA Auditory dysfunction in traumatic brain injury. J Rehabil Res Dev. (2007) 44:921–8. 10.1682/JRRD.2007.09.0140 18075949

[87] Lew HL Garvert DW Pogoda TK Hsu PT Devine JM White DK. Auditory and visual impairments in patients with blast-related traumatic brain injury: effect of dual sensory impairment on functional independence measure. J Rehabil Res Dev. (2009) 46:819–26. 10.1682/JRRD.2008.09.0129 20104405

[88] Lew HL Pogoda TK Baker E Stolzmann KL Meterko M Cifu DX. Prevalence of dual sensory impairment and its association with traumatic brain injury and blast exposure in OEF/OIF veterans. J Head Trauma Rehabil. (2011) 26:489–96. 10.1097/HTR.0b013e318204e54b 21386715

[89] Licona NE Chung JS Poole JH Salerno RM Laurenson NM Harris OA. Prospective tracking and analysis of traumatic brain injury in veterans and military personnel. Arch Phys Med Rehabil. (2017). 98:391–4. 10.1016/j.apmr.2016.09.131 27794484

[90] Lindquist LK Love HC Elbogen EB. Traumatic brain injury in iraq and afghanistan veterans: new results from a national random sample study. J Neuropsychiatry Clin Neurosci. (2017) 29:254–9. 10.1176/appi.neuropsych.16050100 28121256 PMC5501743

[91] Lippa SM Pastorek NJ Benge JF Thornton GM. Postconcussive symptoms after blast and nonblast-related mild traumatic brain injuries in Afghanistan and Iraq war veterans. J Int Neuropsychol Soc. (2010) 16:856–66. 10.1017/S1355617710000743 20682086

[92] Luethcke CA Bryan CJ Morrow CE Isler WC. Comparison of concussive symptoms, cognitive performance, and psychological symptoms between acute blast-versus nonblast-induced mild traumatic brain injury. J Int Neuropsychol Soc. (2011) 17:36–45. 10.1017/S1355617710001207 21083963

[93] MacDonald CL Johnson AM Cooper D Nelson EC Werner NJ Shimony JS. Detection of blast-related traumatic brain injury in U.S. military personnel. N Engl J Med. (2011) 364:2091–100. 10.1056/NEJMoa1008069 21631321 PMC3146351

[94] MacDonald C Johnson A Cooper D Malone T Sorrell J Shimony J Cerebellar white matter abnormalities following primary blast injury in US military personnel. PLoS ONE. (2013) 8:e55823 10.1371/journal.pone.0055823 23409052 PMC3567000

[95] MacDonald CL Johnson AM Nelson EC Werner NJ Fang R Flaherty SF. Functional status after blast-plus-impact complex concussive traumatic brain injury in evacuated United States military personnel. J Neurotrauma. (2014) 31:889–98. 10.1089/neu.2013.3173 24367929 PMC4012688

[96] MacDonald CL Johnson AM Wierzechowski L Kassner E Stewart T Nelson EC Prospectively assessed clinical outcomes in concussive blast vs nonblast traumatic brain injury among evacuated US military personnel. JAMA Neurol. (2014) 71:994–1002. 24934200 10.1001/jamaneurol.2014.1114

[97] MacDonald CL Adam OR Johnson AM Nelson EC Werner NJ Rivet DJ Acute post-traumatic stress symptoms and age predict outcome in military blast concussion. Brain. (2015) 138:1314–26. 25740219 10.1093/brain/awv038 PMC5963403

[98] Mac Donald CL Barber J Andre J Evans N Panks C Sun S. 5-Year imaging sequelae of concussive blast injury and relation to early clinical outcome. Neuroimage Clin. (2017). 14:371–8. 10.1016/j.nicl.2017.02.005 28243574 PMC5320067

[99] Macera CA Aralis HJ MacGregor AJ Rauh MJ Galarneau MR Postdeployment symptom changes and traumatic brain injury and/or posttraumatic stress disorder in men. J Rehabil Res Dev. (2012) 49:1197–208. 10.1682/JRRD.2011.07.0131 23341312

[100] MacGregor AJ Dougherty AL Galarneau MR. Injury-specific correlates of combat-related traumatic brain injury in Operation Iraqi Freedom. J Head Trauma Rehabil. (2011). 26:312–8. 10.1097/HTR.0b013e3181e94404 20808241

[101] MacGregor AJ Dougherty AL Morrison RH Quinn KH Galarneau MR. Repeated concussion among U.S. military personnel during Operation Iraqi Freedom. J Rehabil Res Dev. (2011). 48:1269–78. 10.1682/JRRD.2011.01.0013 22234670

[102] Magone MT Kwon E Shin SY. Chronic visual dysfunction after blast-induced mild traumatic brain injury. J Rehabil Res Dev. (2014) 51:71–80. 10.1682/JRRD.2013.01.0008 24805895

[103] Maguen S Madden E Lau KM Seal K. The impact of head injury mechanism on mental health symptoms in veterans: do number and type of exposures matter? J Trauma Stress. (2012) 25:3–9. 10.1002/jts.21669 22354503

[104] Matthews SC Strigo IA Simmons AN O'connell RM Reinhardt LE Moseley SA. A multimodal imaging study in U.S. veterans of operations iraqi and enduring freedom with and without major depression after blast-related concussion. Neuroimage. (2011) 54:S69–75. 10.1016/j.neuroimage.2010.04.269 20451622

[105] Mendez MF Owens EM Jimenez EE Peppers D Licht EA. Changes in personality after mild traumatic brain injury from primary blast vs. blunt forces. Brain Inj. (2013) 27:10–18. 10.3109/02699052.2012.722252 23252434

[106] Mendez MF Owens EM Reza Berenji G Peppers DC Liang LJ Licht EA. Mild traumatic brain injury from primary blast vs. blunt forces: post-concussion consequences and functional neuroimaging. NeuroRehabilitation. (2013) 32:397–407. 10.3233/NRE-130861 23535805

[107] Miller DR Hayes JP Lafleche G Salat DH Verfaellie M. White matter abnormalities are associated with chronic postconcussion symptoms in blast-related mild traumatic brain injury. Hum Brain Mapp. (2016) 37:220–9. 10.1002/hbm.23022 26497829 PMC4760357

[108] Morey RA Haswell CC Selgrade ES Massoglia D Liu C Weiner J. Effects of chronic mild traumatic brain injury on white matter integrity in Iraq and Afghanistan war veterans. Hum Brain Mapp. (2013) 34:2986–2999. 10.1002/hbm.22117 22706988 PMC3740035

[109] Nathan DE Bellgowan JF Oakes TR French LM Nadar SR Sham EB. Assessing quantitative changes in intrinsic thalamic networks in blast and nonblast mild traumatic brain injury: implications for mechanisms of injury. Brain Connect. (2016) 6:389–402. 10.1089/brain.2015.0403 26956452

[110] Neipert L Pastorek NJ Troyanskaya M Scheibel RS Petersen NJ Levin HS. Effect of clinical characteristics on cognitive performance in service members and veterans with histories of blast-related mild traumatic brain injury. Brain Inj. (2014) 28:1667–74. 10.3109/02699052.2014.947623 25180439

[111] Newsome MR Durgerian S Mourany L Scheibel RS Lowe MJ Beall EB. Disruption of caudate working memory activation in chronic blast-related traumatic brain injury. Neuroimage Clin. (2015) 8:543–53. 10.1016/j.nicl.2015.04.024 26110112 PMC4477106

[112] Norris JN Sams R Lundblad P Frantz E Harris E. Blast-related mild traumatic brain injury in the acute phase: acute stress reactions partially mediate the relationship between loss of consciousness and symptoms. Brain Inj. (2014) 28:1052–62. 10.3109/02699052.2014.891761 24655334

[113] Norris JN Smith S Harris E Labrie DW Ahlers ST. Characterization of acute stress reaction following an IED blast-related mild traumatic brain injury. Brain Inj. (2015) 29:898–904. 10.3109/02699052.2015.1022879 25955118

[114] Oleksiak M Smith BM St. Andre JR Caughlan CM Steiner M. Audiological issues and hearing loss among veterans with mild traumatic brain injury. J Rehabil Res Dev. (2012) 49:995–1004. 10.1682/JRRD.2011.01.0001 23341275

[115] O'Neil ME Callahan M Carlson KF Roost M Laman-Maharg B Twamley EW. Postconcussion symptoms reported by operation enduring freedom/operation iraqi freedom veterans with and without blast exposure, mild traumatic brain injury, and posttraumatic stress disorder. J Clin Exp Neuropsychol. (2017) 39:449–58. 10.1080/13803395.2016.1232699 27681407

[116] Petrie EC Cross DJ Yarnykh VL Richards T Martin NM Pagulayan K. Neuroimaging, behavioral, and psychological sequelae of repetitive combined blast/impact mild traumatic brain injury in Iraq and afghanistan war veterans. J Neurotrauma. (2014) 31:425–36. 10.1089/neu.2013.2952 24102309 PMC3934596

[117] Pogoda TK Hendricks AM Iverson KM Stolzmann KL Krengel MH Baker E. Multisensory impairment reported by veterans with and without mild traumatic brain injury history. J Rehabil Res Dev. (2012) 49:971–84. 10.1682/JRRD.2011.06.0099 23341273

[118] Reid MW Miller KJ Lange RT Cooper DB Tate DF Bailie J. A multisite study of the relationships between blast exposures and symptom reporting in a post-deployment active duty military population with mild traumatic brain injury. J Neurotrauma. (2014) 31:1899–906. 10.1089/neu.2014.3455 25036531 PMC4840828

[119] Riedy G Senseney JS Liu W Ollinger J Sham E Krapiva P. Findings from structural MR imaging in military traumatic brain injury. Radiology. (2016) 279:207–15. 10.1148/radiol.2015150438 26669604

[120] Robinson ME Lindemer ER Fonda JR Milberg WP Mcglinchey RE Salat DH. Close-range blast exposure is associated with altered functional connectivity in Veterans independent of concussion symptoms at time of exposure. Hum Brain Map. (2015) 36:911–22. 10.1002/hbm.22675 25366378 PMC6869346

[121] Ruff RL Ruff SS Wang XF. Headaches among operation iraqi freedom/operation enduring freedom veterans with mild traumatic brain injury associated with exposures to explosions. J Rehabil Res Dev. (2008) 45:941–52. 10.1682/JRRD.2008.02.0028 19165684

[122] Ruff RL Ruff SS Wang XF. Improving sleep: initial headache treatment in OIF/OEF veterans with blast-induced mild traumatic brain injury. J Rehabil Res Dev. (2009) 46:1071–84. 10.1682/JRRD.2009.05.0062 20437313

[123] Ryu J Horkayne-Szakaly I Xu L Pletnikova O Leri F Eberhart C. The problem of axonal injury in the brains of veterans with histories of blast exposure. Acta Neuropathol Commun. (2014) 2:153. 10.1186/s40478-014-0153-3 25422066 PMC4260204

[124] Saxe JL Perdue CL. Associations between operationally estimated blast exposures and postdeployment diagnoses of postconcussion syndrome and posttraumatic stress disorder. US Army Med Dep J. (2015) 73–78. 25651149

[125] Scheibel RS Newsome MR Troyanskaya M Lin X Steinberg JL Radaideh M Altered brain activation in military personnel with one or more traumatic brain injuries following blast. J Int Neuropsychol Soc. (2012) 18:89–100. 10.1017/S1355617711001433 22132942

[126] Storzbach D O'Neil ME Roost SM Kowalski H Iverson GL. Comparing the neuropsychological test performance of Operation Enduring Freedom/Operation Iraqi Freedom (OEF/OIF) veterans with and without blast exposure, mild traumatic brain injury, and posttraumatic stress symptoms. J Int Neuropsychol Soc. (2015) 21:353–63. 10.1017/S1355617715000326 26029852

[127] Stout J Bolzenius J Roskos PT Osman M Frausto R Bucholz R FMRI assessment of load based working memory in blast TBI and associated effects in FDG-PET signal. In: International IEEE/EMBS Conference on Neural Engineering. (2013) p. 770–3. 10.1109/NER.2013.6696048

[128] Strigo IA Spadoni AD Lohr J Simmons AN. Too hard to control: compromised pain anticipation and modulation in mild traumatic brain injury. Transl Psychiatry. (2014) 4:e340. 10.1038/tp.2013.116 24399043 PMC3905226

[129] Tate DF York GE Reid MW Cooper DB Jones L Robin DA. Preliminary findings of cortical thickness abnormalities in blast injured service members and their relationship to clinical findings. Brain Imaging Behav. (2014) 8:102–9. 10.1007/s11682-013-9257-9 24100952 PMC4714342

[130] Trotter BB Robinson ME Milberg WP Mcglinchey RE Salat DH. Military blast exposure, ageing and white matter integrity. Brain. (2015) 138:2278–2292. 10.1093/brain/awv139 26033970 PMC4840948

[131] Troyanskaya M Pastorek NJ Scheibel RS Petersen NJ McCulloch K Wilde EA. Combat exposure, PTSD symptoms, and cognition following blast-related traumatic brain injury in OEF/OIF/OND service members and Veterans. Mil Med. (2015) 180:285–9. 10.7205/MILMED-D-14-00256 25735018

[132] Trudeau DL Anderson J Hansen LM Shagalov DN Schmoller J Nugent S. Findings of mild traumatic brain injury in combat veterans with PTSD and a history of blast concussion. J Neuropsychiatry Clin Neurosci. (1998) 10:308–13. 10.1176/jnp.10.3.308 9706538

[133] Vakhtin AA Calhoun VD Jung RE Prestopnik JL Taylor PA Ford CC. Changes in intrinsic functional brain networks following blast-induced mild traumatic brain injury. Brain Inj. (2013) 27:1304–10. 10.3109/02699052.2013.823561 24020442 PMC5075489

[134] Verfaellie M Lafleche G Spiro A 3rd Tun C Bousquet K. Chronic postconcussion symptoms and functional outcomes in OEF/OIF veterans with self-report of blast exposure. J. Int. Neuropsychol. Soc. (2013) 19:1–10. 10.1017/S1355617712000902 23095177

[135] Verfaellie M Lee LO Lafleche G Spiro A. Self-reported sleep disturbance mediates the relationship between PTSD and cognitive outcome in blast-exposed OEF/OIF veterans. J Head Trauma Rehabil. (2016) 31:309–19. 10.1097/HTR.0000000000000197 26580692 PMC4870155

[136] Walsh DV Capó-Aponte JE Jorgensen-Wagers K Temme LA Goodrich G Sosa J. Visual field dysfunctions in warfighters during different stages following blast and nonblast mTBI. Mil Med. (2014) 180:178–85. 10.7205/MILMED-D-14-00230 25643385

[137] Wares JR Hoke KW Walker W Franke LM Cifu DX Carne W. Characterizing effects of mild traumatic brain injury and posttraumatic stress disorder on balance impairments in blast-exposed servicemembers and veterans using computerized posturography. J Rehabil Res Dev. (2015) 52:591–603. 10.1682/JRRD.2014.08.0197 26437003

[138] Wilk JE Thomas JL Mcgurk DM Riviere LA Castro CA Hoge CW. Mild traumatic brain injury (concussion) during combat: lack of association of blast mechanism with persistent postconcussive symptoms. J Head Trauma Rehabil. (2010) 25:9–14. 10.1097/HTR.0b013e3181bd090f 20051900

[139] Wilkinson CW Pagulayan KF Petrie EC Mayer CL Colasurdo EA Shofer JB. High prevalence of chronic pituitary and target-organ hormone abnormalities after blast-related mild traumatic brain injury. Front Neurol. (2012) 3:11. 10.3389/fneur.2012.00011 22347210 PMC3273706

[140] Xydakis MS Mulligan LP Smith AB Olsen CH Lyon DM Belluscio L. Olfactory impairment and traumatic brain injury in blast-injured combat troops: a cohort study. Neurology. (2015) 84:1559–67. 10.1212/WNL.0000000000001475 25788559 PMC4408285

[141] Yeh PH Wang B Oakes TR French LM Pan H Graner J. Postconcussional disorder and PTSD symptoms of military-related traumatic brain injury associated with compromised neurocircuitry. Hum Brain Mapp. (2014) 35:2652–73. 10.1002/hbm.22358 24038816 PMC6869078

[142] Yeh PH Guan Koay C Wang B Morissette J Sham E Senseney J. Compromised neurocircuitry in chronic blast-related mild traumatic brain injury. Hum Brain Mapp. (2017) 38:352–69. 10.1002/hbm.23365 27629984 PMC6867097

[143] Greer N Sayer N Kramer M Koeller E Velasquez T. Prevalence and Epidemiology of Combat Blast Injuries from the Military Cohort 2001-2014. Washington, DC: Department of Veterans Affairs (US) (2016). 28813129

[144] Carroll LJ Cassidy JD Peloso PM Borg J Von Holst H Holm L Prognosis for mild traumatic brain injury: results of the WHO collaborating centre task force on mild traumatic brain injury. J Rehabil Med. (2004) (43 Suppl)84–105. 10.1080/16501960410023859 15083873

[145] Nelson NW Davenport ND Sponheim SR Anderson CR Blast-related mild traumatic brain injury: neuropsychological evaluation and findings. In: Kobeissy FH editor. Brain Neurotrauma: Molecular, Neuropsychological, Rehabilitation Aspects. Boca Raton, FL (2015). p. 451–70. 10.1201/b18126-38 26269927

[146] Cole WR Bailie JM Neurocognitive psychiatric symptoms following mild traumatic brain injury. In: Laskowitz D Grant G editors. Translational Research in Traumatic Brain Injury. Boca Raton, FL (2016). p. 1–38. 10.1201/b18959-20 26583174

[147] Iverson GL Outcome from mild traumatic brain injury. Curr Opin Psychiatry. (2005) 18:301–17. 10.1097/01.yco.0000165601.29047.ae 16639155

[148] Hoffman SW DePalma RG Cifu Dx. Veterans affairs traumatic brain injury conference: state of the art. Brain Inj. (2017) 31:1165–7. 10.1080/02699052.2017.1359336 28981344

[149] Weiskopf NG Weng C. Methods and dimensions of electronic health record data quality assessment: enabling reuse for clinical research. J Am Med Inform Assoc. (2013) 20:144–51. 10.1136/amiajnl-2011-000681 22733976 PMC3555312

[150] Synnot A Bragge P Lunny C Menon D Clavisi O Pattuwage L. The currency, completeness and quality of systematic reviews of acute management of moderate to severe traumatic brain injury: a comprehensive evidence map. PLoS ONE. (2018) 13:e0198676. 10.1371/journal.pone.0198676 29927963 PMC6013193

[151] Greer N Sayer N Koeller E Velasquez T Wilt TJ. Outcomes associated with blast versus nonblast-related traumatic brain injury in US military service members and veterans: a systematic review. J Head Trauma Rehabil. (2018) 33:E16–E29. 10.1097/HTR.0000000000000304 28422897

[152] Orman JA Geyer D Jones J Schneider EB Grafman J Pugh MJ. Epidemiology of moderate-to-severe penetrating versus closed traumatic brain injury in the Iraq and afghanistan wars. J Trauma Acute Care Surg. (2012) 73:S496–502. 10.1097/TA.0b013e318275473c 23192076

[153] Rohling ML Binder LM Demakis GJ Larrabee GJ Ploetz DM Langhinrichsen-Rohling J. A meta-analysis of neuropsychological outcome after mild traumatic brain injury: re-analyses and reconsiderations of Binder et al. (1997), Frencham et al. (2005), and Pertab et al. (2009). Clin Neuropsychol. 25:608–23. 10.1080/13854046.2011.565076 21512956

[154] Marquez De La Plata CD Hart T Hammond FM Frol AB Hudak A Harper CR. Impact of age on long-term recovery from traumatic brain injury. Arch Phys Med Rehabil. (2008) 89:896–903. 10.1016/j.apmr.2007.12.030 18452739 PMC2600417

[155] Rasmussen TE Elster EA Rauch TM Brix KA. A perspective on the 2014 institute of medicine report on the long-term effects of blast exposures. J Trauma Acute Care Surg. (2014) 77:S237–9. 10.1097/TA.0000000000000383 25159360

[156] Agoston D Arun P Bellgowan P Broglio S Cantu R Cook D. Military blast injury and chronic neurodegeneration: research presentations from the 2015 international state-of-the-science meeting. J Neurotrauma. (2017) 34:S6–S17. 10.1089/neu.2017.5220 28937955 PMC5695753

[157] Brix K Brody DL Grimes JB Yitzhak A Working Group Members Military blast exposure and chronic neurodegeneration: summary of working groups and expert panel findings and recommendations. J Neurotrauma. (2017) 34:S18–25. 10.1089/neu.2017.5222

[158] Leggieri MJ Jr Gupta RK Hinds SR 2nd. International state-of-the-science meeting exploring the potential relationship between blast-related trauma and the development of chronic traumatic encephalopathy. J Neurotrauma. (2017) 34:S1–S3. 10.1089/neu.2017.29013.introduction 28937956

[159] Badea A Kamnaksh A Anderson RJ Calabrese E Long JB Agoston DV. Repeated mild blast exposure in young adult rats results in dynamic and persistent microstructural changes in the brain. Neuroimage Clin. (2018) 18:60–73. 10.1016/j.nicl.2018.01.007 29868442 PMC5984602

[160] RAND The neurological effects of repeated exposure to military occupational blast: implications for prevention and health. In: Proceedings, Findings, and Expert Recommendations from the Seventh Department of Defense State-of-the-Science Meeting. Santa Monica, CA: RAND Corporation, 2019 (2019). Available online at: https://www.rand.org/pubs/conf_proceedings/CF380z1.html (accessed April 19, 2020).

[161] Patten E Parker K Women in the U.S. Military: Growing Share, Distinctive Profile. Washington, DC: Pew Research Center (2011).

[162] Amoroso T Iverson KM. Acknowledging the risk for traumatic brain injury in women veterans. J Nerv Ment Dis. (2017) 205:318–23. 10.1097/NMD.0000000000000621 28350782

[163] Fear NT Jones E Groom M Greenberg N Hull L Hodgetts TJ. Symptoms of post-concussional syndrome are non-specifically related to mild traumatic brain injury in UK armed forces personnel on return from deployment in Iraq: an analysis of self-reported data. Psychol Med. (2009) 39:1379–87. 10.1017/S0033291708004595 18945380

[164] Iverson KM Pogoda TK Gradus JL Street AE. Deployment-related traumatic brain injury among operation enduring freedom/operation iraqi freedom veterans: associations with mental and physical health by gender. J Womens Health. (2013) 22:267–75. 10.1089/jwh.2012.3755 23421581

[165] Brickell TA Lippa SM French LM Kennedy JE Bailie JM Lange RT. Female service members and symptom reporting after combat and non-combat-related mild traumatic brain injury. J Neurotrauma. (2017) 34:300–12. 10.1089/neu.2016.4403 27368356

[166] Lange RT Brickell TA Kennedy JE Bailie JM Sills C Asmussen S. Factors influencing postconcussion and posttraumatic stress symptom reporting following military-related concurrent polytrauma and traumatic brain injury. Arch Clin Neuropsychol. (2014) 29:329–47. 10.1093/arclin/acu013 24723461

[167] Lippa SM Brickell TA Bailie JM French LM Kennedy JE Lange RT. Postconcussion symptom reporting after mild traumatic brain injury in female service members: impact of gender, posttraumatic stress disorder, severity of injury, and associated bodily injuries. J Head Trauma Rehabil. (2018) 33:101–12. 10.1097/HTR.0000000000000353 29084103

[168] Chappell B Pentagon Says Women Can Now Serve in Front-Line Ground Combat Positions. NPR (2015). Available online at: https://www.npr.org/sections/thetwo-way/2015/12/03/458319524/pentagon-will-allow-women-in-frontline-ground-combat-positions (accessed January 13, 2020).

[169] Wells TS Seelig AD Ryan MA Jones JM Hooper TI Jacobson IG. Hearing loss associated with US military combat deployment. Noise Health. (2015) 17:34–42. 10.4103/1463-1741.149574 25599756 PMC4918647

[170] Lawson BD Kass SJ Dhillon KK Milam LS Cho TH Rupert AH. Military occupations most affected by head/sensory injuries and the potential job impact of those injuries. Mil Med. (2016) 181:887–94. 10.7205/MILMED-D-15-00184 27483529

[171] Nelson JT Swan AA Swiger B Packer M Pugh MJ. Hearing testing in the U.S. department of defense: potential impact on veterans affairs hearing loss disability awards. Hear Res. (2017) 349:13–20. 10.1016/j.heares.2016.10.005 27768901

[172] Administration VH Veterans! Hard of Hearing? VA Can Help. (2015) Available online at: https://www.va.gov/HEALTH/NewsFeatures/2015/September/Veterans-Hard-of-Hearing-VA-Can-Help.asp (accessed August 21, 2019).

[173] Singman E Quaid P Vision disorders in mild traumatic brain injury. In: Blaban MHAC editor. Neurosensory Disorders in Mild Traumatic Brain Injury. London: Elsevier (2019). p. 223–44. 10.1016/B978-0-12-812344-7.00015-7

[174] Patil VK St. Andre JR Crisan E Smith BM Evans CT Steiner ML. Prevalence and treatment of headaches in veterans with mild traumatic brain injury. Headache. (2011) 51:1112–21. 10.1111/j.1526-4610.2011.01946.x 21762135

[175] Guitera V Munoz P Castillo J Pascual J. Quality of life in chronic daily headache: a study in a general population. Neurology. (2002) 58:1062–5. 10.1212/WNL.58.7.1062 11940693

[176] Iverson KM Hendricks AM Kimerling R Krengel M Meterko M Stolzmann KL. Psychiatric diagnoses and neurobehavioral symptom severity among OEF/OIF VA patients with deployment-related traumatic brain injury: a gender comparison. Womens Health Issues. (2011) 21:S210–17. 10.1016/j.whi.2011.04.019 21724143 PMC3132395

[177] Franke LM Czarnota JN Ketchum JM Walker WC. Factor analysis of persistent postconcussive symptoms within a military sample with blast exposure. J Head Trauma Rehabil. (2015) 30:E34–46. 10.1097/HTR.0000000000000042 24695267 PMC4286462

[178] Smith BM Evans CT Mild Traumatic Brain Injury: Screening and Comorbidities. Forum: Translating Research into Quality Healthcare for Veterans (2013).

[179] Geiling J Rosen JM Edwards RD. Medical costs of war in 2035: long-term care challenges for veterans of Iraq and afghanistan. Mil Med. (2012) 177:1235–44. 10.7205/MILMED-D-12-00031 23198496

[180] Dismuke-Greer C Hirsch S Carlson K Pogoda T Nakase-Richardson R Bhatnagar S Health services utilization, health care costs, and diagnoses by mild traumatic brain injury exposure: a chronic effects of neurotrauma consortium study. Arch Phys Med Rehabil. (2020) 101:1720–30. 10.1016/j.apmr.2020.06.008 32653582

[181] Jaycox L Tanielian T Invisible wounds of war: psychological and cognitive injuries, their consequences, and services to assist recovery. Santa Monica, CA: RAND Corporation, Center for Military Health Policy Research (2008). 10.1037/e527612010-001

[182] CDMRP Transforming Healthcare through Innovative and Impactful Research. Ft Detrick, MD (2020). Available online at: https://cdmrp.army.mil (accessed April 19, 2020).

[183] Nichols GA Bell TJ Pedula KL O'keeffe-Rosetti M. Medical care costs among patients with established cardiovascular disease. Am J Manag Care. (2010) 16:e86–e93. 20205493

[184] Majdan M Plancikova D Maas A Polinder S Feigin V Theadom A. Years of life lost due to traumatic brain injury in Europe: a cross-sectional analysis of 16 countries. PLoS Med. (2017) 14:e1002331. 10.1371/journal.pmed.1002331 28700588 PMC5507416

[185] Bryden DW Tilghman JI Hinds SR II. Blast-related traumatic brain injury: current concepts and research considerations. J Exp Neurosci. (2019) 13:1179069519872213. 10.1177/1179069519872213 31548796 PMC6743194

[186] Roozenbeek B Maas AI Menon DK. Changing patterns in the epidemiology of traumatic brain injury. Nat Rev Neurol. (2013) 9:231–6. 10.1038/nrneurol.2013.22 23443846

[187] GBD 2016 Traumatic Brain Injury and Spinal Cord Injury Collaborators. Global, regional, and national burden of traumatic brain injury and spinal cord injury, 1990-2016: a systematic analysis for the global burden of disease study 2016. Lancet Neurol. (2019) 18:56–87. 10.1016/S1474-4422(18)30415-0 30497965 PMC6291456

[188] Tadisina KK Abcarian A Omi E. Facial firework injury: a case series. West J Emerg Med. (2014) 15:387–93. 10.5811/westjem.2014.1.19857 25035740 PMC4100840

[189] Smith LGD Dornbos D Leonard J Traumatic subdural hematoma and intraparenchymal contusion after a firework blast injury. J Head Neck Spine Surg. (2019) 4:555627 10.19080/JHNSS.2019.04.555627

[190] Carlozzi NE Lange RT French LM Sander AM Freedman J Brickell TA. A latent content analysis of barriers and supports to healthcare: perspectives from caregivers of service members and veterans with military-related traumatic brain injury. J Head Trauma Rehabil. (2018) 33:342–53. 10.1097/HTR.0000000000000373 29385014 PMC6172008

[191] Francia K A Case Study on the Army Field of Helmet Sensors and Blast Gauges. Monterey, CA: Naval Postgraduate School (2018).

[192] Olenick M Flowers M Diaz VJ. US veterans and their unique issues: enhancing health care professional awareness. Adv Med Educ Pract. (2015) 6:635–9. 10.2147/AMEP.S89479 26664252 PMC4671760

[193] Hamilton J Army 'Leans In' To Protect A Shooter's Brain From Blast Injury. The Impact of *War* (2018). Available online at: https://www.npr.org/sections/health-shots/2018/05/17/611700850/army-takes-steps-to-protect-shooters-brain-from-blast-injury/ (accessed April 19, 2020).

[194] Weapons DSBTFODE Final Report of the Defense Science Board Task Force on Directed Energy Weapon Systems and Technology Applications. T. Office of the under Secretary of Defense for Acquisition, and Logistics, editor. Washington, DC (2007).

[195] Jin H Hou LJ Wang ZG. Military brain science - how to influence future wars. Chin J Traumatol. (2018) 21:277–80. 10.1016/j.cjtee.2018.01.006 30279039 PMC6235785

