# Supplementary material for: Characteristics and Impact of U.S. Military Blast-Related Mild Traumatic Brain Injury: A Systematic Review
Source: Front Neurol. 2020 Nov 2;11:559318. doi: 10.3389/fneur.2020.559318 (PMC7667277; doi:10.3389/fneur.2020.559318)
Supplement: Supplementary file 1 [file Data_Sheet_1.PDF]

Database: Ovid MEDLINE(R) Epub Ahead of Print, In-Process & Other Non-Indexed Citations, Ovid MEDLINE(R) Daily and Ovid MEDLINE(R) <1946 to Present>
Search Strategy:
----
1 exp Blast Injuries/ (3744)
2 exp Explosions/ (3680)
3 (blast* or explosion* or detonat*).mp. [mp=title, abstract, original title, name of substance word, subject heading word, keyword heading word, protocol supplementary concept word, rare disease supplementary concept word, unique identifier, synonyms] (101028)
4 exp "United States Department of Veterans Affairs"/ (6422)
5 exp Veterans Health/ (745)
6 exp Veterans/ (13029)
7 exp Veterans Disability Claims/ (276)
8 exp Military Medicine/ (28451)
9 exp "United States Department of Defense"/ (247)
10 exp Naval Medicine/ (9455)
11 exp Military Facilities/ (152)
12 exp Military Personnel/ (35320)
13 exp hospitals, military/ (4952)
14 exp hospitals, veterans/ (6315)
15 exp afghan campaign 2001-/ (2191)
16 exp gulf war/ (379)
17 exp iraq war, 2003-2011/ (2484)
18 exp war exposure/ (234)
19 (military* or veteran* or ((army or armed) adj3 force*) or armies or navy or marine* or soldier* or OIF or OEF or deployment or (service adj3 member*) or (operation* adj3 (freedom or Dawn)) or war or wars or afghan* or Iraq* or gulf).mp. [mp=title, abstract, original title, name of substance word, subject heading word, keyword heading word, protocol supplementary concept word, rare disease supplementary concept word, unique identifier, synonyms] (244678)
20 exp Brain Injuries/ (58629)
21 TBI*.mp. (20885)
22 ((brain* or encephalon* or cranium or cranio* or cranial or intracrani* or intra-crani* or skull* or cerebral or cerebell* or head or ventric* or pontine or putamin* or dura* or subdura* or sub-dura* or ((supra or extra) adj2 dura*) or supradura* or epidura* or epi-dura* or arachnoid* or sub-arachnoid* or subarachnoid* or (intra adj2 arachnoid*)) adj3 (injur* or trauma* or concussion* or post-concussion* or h?emorrhag* or h?ematom* or bleed* or penetrat* or (non adj2 penetrat*) or edema* or oedema* or fracture* or aneurysm* or pressur*)).mp. [mp=title, abstract, original title, name of substance word, subject heading word, keyword heading word, protocol supplementary concept word, rare disease supplementary concept word, unique identifier, synonyms] (291210)
23 Neurosurgery/ or exp craniotomy/ or decompressive craniectomy/ or trephining/ (26900)
24 (neurosurg* or neuro-surg* or craniotom* or craniectom* or trepanation* or trepanning* or trephination* or trephining*).mp. [mp=title, abstract, original title, name of substance word, subject heading word, keyword heading word, protocol supplementary concept word, rare disease supplementary concept word, unique identifier, synonyms] (82765)
25 brain injury, chronic/ or brain edema/ or exp brain concussion/ or contrecoup injury/ or post-concussion syndrome/ or exp brain hemorrhage, traumatic/ or brain stem hemorrhage, traumatic/ or cerebral hemorrhage, traumatic/ or pneumocephalus/ or exp intracranial hemorrhages/ or exp cerebral hemorrhage/ or putaminal hemorrhage/ or exp intracranial hemorrhage, hypertensive/ or exp intracranial hemorrhage, traumatic/ or exp hematoma, epidural, cranial/ or exp hematoma, subdural/ or hematoma, subdural, acute/ or hematoma, subdural, chronic/ or hematoma, subdural, intracranial/ or subarachnoid hemorrhage, traumatic/ or exp pituitary apoplexy/ or exp subarachnoid hemorrhage/ or exp intracranial hypertension/ or exp trauma, nervous system/ or cerebrospinal fluid leak/ or cerebrospinal fluid otorrhea/ or cerebrospinal fluid rhinorrhea/ or coma, post-head injury/ or exp cranial nerve injuries/ or exp head injuries, closed/ or head injuries, penetrating/ or exp skull fractures/ or skull fracture, basilar/ or skull fracture, depressed/ or exp Cerebrovascular Trauma/ (247975)

26 (pneumocephal* or pneumo-cephal* or a?rocele* or pneumocyst* or pneumo-cyst* or ((CSF or cerebrospinal) adj3 (leak* or otorrhea* or rhinorrhea*)) or co?ntrecoup* or (co?ntre adj3 coup*)).mp. [mp=title, abstract, original title, name of substance word, subject heading word, keyword heading word, protocol supplementary concept word, rare disease supplementary concept word, unique identifier, synonyms] (23299)
27 1 or 2 or 3 (101028)
28 4 or 5 or 6 or 7 or 8 or 9 or 10 or 11 or 12 or 13 or 14 or 15 or 16 or 17 or 18 or 19 (248463)
29 20 or 21 or 22 or 23 or 24 or 25 or 26 (465755)
30 27 and 28 and 29 (922)

****

Recent queries in pubmed

Search	Query	Items found	Time
#56	Search #53 AND #54 AND #55	1020	9:54:53
#55	Search #20 OR #21 OR #22 OR #23 OR #24 OR #25 OR #26 OR #27 OR #28 OR #29 OR #30 OR #31 OR #32 OR #33 OR #34 OR #35 OR #36 OR #37 OR #38 OR #39 OR #40 OR #41 OR #42 OR #43 OR #44 OR #45 OR #46 OR #47 OR #48 OR #48 OR #50 OR #51 OR #52	462811	9:53:58
#54	Search #4 OR #5 OR #6 OR #7 OR #8 OR #9 OR #10 OR #11 OR #12 OR #13 OR #14 OR #15 OR #16 OR #17 OR #18 OR #19	480679	9:51:12
#53	Search #1 OR #2 OR #3	85385	9:48:45
#52	Search pneumocephal*[tw] OR pneumo-cephal*[tw] OR aerocel*[tw] OR pneumocyst*[tw] OR pneumo-cyst*[tw] OR CSF leak*[tw] OR CSF Otorrhea*[tw] OR CSF rhinorrhea*[tw] OR cerebrospinal fluid leak*[tw] OR cerebrospinal fluid Otorrhea*[tw] OR cerebrospinal fluid rhinorrhea*[tw] OR cerebro-spinal fluid leak*[tw] OR cerebro-spinal fluid Otorrhea*[tw] OR cerebro-spinal fluid rhinorrhea*[tw] OR contrecoup* OR countercoup*[tw] OR contre-coup*[tw] OR counter-coup*[tw]	22457	9:47:28
#51	Search neurosurg*[tw] OR neuro-surg*[tw] OR craniotomy*[tw] OR craniectom*[tw] OR decompressive craniectom*[tw] OR trephin*[tw] OR trepanation*[tw] OR trepan*[tw] OR trephine*[tw]	83531	9:47:10
#50	Search brain injury, chronic [mesh:noexp] OR brain edema [mesh:noexp] OR brain concussion [mesh] OR contrecoup injury [mesh:noexp] OR post-concussion syndrome [mesh:noexp] OR brain hemorrhage, traumatic [mesh] OR brain stem hemorrhage, traumatic [mesh:noexp] OR cerebral hemorrhage, traumatic [mesh:noexp] OR pneumocephalus [mesh:noexp] OR intracranial hemorrhages [mesh] OR cerebral hemorrhage [mesh] OR putaminal hemorrhage [mesh:noexp] OR intracranial hemorrhage, hypertensive [mesh] OR intracranial hemorrhage, traumatic [mesh] OR hematoma, epidural, cranial [mesh] OR hematoma, subdural [mesh] OR hematoma, subdural, acute [mesh:noexp] OR hematoma, subdural, chronic [mesh:noexp] OR hematoma, subdural, intracranial [mesh:noexp] OR subarachnoid hemorrhage, traumatic [mesh:noexp] OR pituitary apoplexy [mesh] OR subarachnoid hemorrhage [mesh] OR intracranial hypertension [mesh] OR trauma, nervous system [mesh] OR cerebrospinal fluid leak [mesh:noexp] OR cerebrospinal fluid otorrhea [mesh:noexp] OR cerebrospinal fluid rhinorrhea [mesh:noexp] OR coma, post-head injury [mesh:noexp] OR cranial nerve injuries [mesh] OR head injuries, closed [mesh] OR head injuries, penetrating [mesh:noexp] OR skull fractures [mesh] OR skull fracture, basilar [mesh:noexp] OR skull fracture, depressed [mesh:noexp] OR Cerebrovascular Trauma [mesh]	243669	9:47:03
#49	Search neurosurgery [mesh:noexp] OR craniotomy [mesh] OR decompressive craniectomy [mesh:noexp] OR trephining [mesh:noexp]	46540	9:46:00

#48	Search sub-arachnoidal injur*[tw] OR sub-arachnoidal traum*[tw] OR sub-arachnoidal concussion*[tw] OR sub-arachnoidal postconcussion*[tw] OR sub-arachnoidal post-concussion*[tw] OR sub-arachnoidal hemorrhag*[tw] OR sub-arachnoidal haemorrhag*[tw] OR sub-arachnoidal hematom*[tw] OR sub-arachnoidal bleed*[tw] OR sub-arachnoidal penetrat*[tw] OR sub-arachnoidal nonpenetrat*[tw] OR sub-arachnoidal non-penetrat*[tw]	7	9:45:40
#47	Search sub-arachnoid injur*[tw] OR sub-arachnoid traum*[tw] OR sub-arachnoid concussion*[tw] OR sub-arachnoid postconcussion*[tw] OR sub-arachnoid post-concussion*[tw] OR sub-arachnoid hemorrhag*[tw] OR sub-arachnoid haemorrhag*[tw] OR sub-arachnoid hematom*[tw] OR sub-arachnoid bleed*[tw] OR sub-arachnoid penetrat*[tw] OR sub-arachnoid nonpenetrat*[tw] OR sub-arachnoid non-penetrat*[tw]	140	9:45:24
#46	Search subarachnoidal injur*[tw] OR subarachnoidal traum*[tw] OR subarachnoidal concussion*[tw] OR subarachnoidal postconcussion*[tw] OR subarachnoidal post-concussion*[tw] OR subarachnoidal hemorrhag*[tw] OR subarachnoidal haemorrhag*[tw] OR subarachnoidal hematom*[tw] OR subarachnoidal bleed*[tw] OR subarachnoidal penetrat*[tw] OR subarachnoidal nonpenetrat*[tw] OR subarachnoidal non-penetrat*[tw]	2249	9:44:32
#45	Search subarachnoid injur*[tw] OR subarachnoid traum*[tw] OR subarachnoid concussion*[tw] OR subarachnoid postconcussion*[tw] OR subarachnoid post-concussion*[tw] OR subarachnoid hemorrhag*[tw] OR subarachnoid haemorrhag*[tw] OR subarachnoid hematom*[tw] OR subarachnoid bleed*[tw] OR subarachnoid penetrat*[tw] OR subarachnoid nonpenetrat*[tw] OR subarachnoid non-penetrat*[tw]	27896	9:44:15
#44	Search arachnoidal injur*[tw] OR arachnoidal traum*[tw] OR arachnoidal concussion*[tw] OR arachnoidal postconcussion*[tw] OR arachnoidal post-concussion*[tw] OR arachnoidal hemorrhag*[tw] OR arachnoidal haemorrhag*[tw] OR arachnoidal hematom*[tw] OR arachnoidal bleed*[tw] OR arachnoidal penetrat*[tw] OR arachnoidal nonpenetrat*[tw] OR arachnoidal non-penetrat*[tw]	103	9:43:55
#43	Search arachnoid injur*[tw] OR arachnoid traum*[tw] OR arachnoid concussion*[tw] OR arachnoid postconcussion*[tw] OR arachnoid post-concussion*[tw] OR arachnoid hemorrhag*[tw] OR arachnoid haemorrhag*[tw] OR arachnoid hematom*[tw] OR arachnoid bleed*[tw] OR arachnoid penetrat*[tw] OR arachnoid nonpenetrat*[tw] OR arachnoid non-penetrat*[tw]	1545	9:43:11
#42	Search epi-dural injur*[tw] OR epi-dural traum*[tw] OR epi-dural concussion*[tw] OR epi-dural postconcussion*[tw] OR epi-dural post-concussion*[tw] OR epi-dural hemorrhag*[tw] OR epi-dural haemorrhag*[tw] OR epi-dural hematom*[tw] OR epi-dural bleed*[tw] OR epi-dural penetrat*[tw] OR epi-dural nonpenetrat*[tw] OR epi-dural non-penetrat*[tw]	2	9:42:56

#41	Search epidural injur*[tw] OR epidural traum*[tw] OR epidural concussion*[tw] OR epidural postconcussion*[tw] OR epidural post-concussion*[tw] OR epidural hemorrhag*[tw] OR epidural haemorrhag*[tw] OR epidural hematom*[tw] OR epidural bleed*[tw] OR epidural penetrat*[tw] OR epidural nonpenetrat*[tw] OR epidural non-penetrat*[tw]	8809	9:42:39
#40	Search extra-dural injur*[tw] OR extra-dural traum*[tw] OR extra-dural concussion*[tw] OR extra-dural postconcussion*[tw] OR extra-dural post-concussion*[tw] OR extra-dural hemorrhag*[tw] OR extra-dural haemorrhag*[tw] OR extra-dural hematom*[tw] OR extra-dural bleed*[tw] OR extra-dural penetrat*[tw] OR extra-dural nonpenetrat*[tw] OR extra-dural non-penetrat*[tw]	38	9:42:24
#39	Search extradural injur*[tw] OR extradural traum*[tw] OR extradural concussion*[tw] OR extradural postconcussion*[tw] OR extradural post-concussion*[tw] OR extradural hemorrhag*[tw] OR extradural haemorrhag*[tw] OR extradural hematom*[tw] OR extradural bleed*[tw] OR extradural penetrat*[tw] OR extradural nonpenetrat*[tw] OR extradural non-penetrat*[tw]	1497	9:42:12
#38	Search supradural injur*[tw] OR supradural traum*[tw] OR supradural concussion*[tw] OR supradural postconcussion*[tw] OR supradural post-concussion*[tw] OR supradural hemorrhag*[tw] OR supradural haemorrhag*[tw] OR supradural hematom*[tw] OR supradural bleed*[tw] OR supradural penetrat*[tw] OR supradural nonpenetrat*[tw] OR supradural non-penetrat*[tw]	4	9:41:53
#37	Search sub-dural injur*[tw] OR sub-dural traum*[tw] OR sub-dural concussion*[tw] OR sub-dural postconcussion*[tw] OR sub-dural post-concussion*[tw] OR sub-dural hemorrhag*[tw] OR sub-dural haemorrhag*[tw] OR sub-dural hematom*[tw] OR sub-dural bleed*[tw] OR sub-dural penetrat*[tw] OR sub-dural nonpenetrat*[tw] OR sub-dural non-penetrat*[tw]	39	9:41:39
#36	Search subdural injur*[tw] OR subdural traum*[tw] OR subdural concussion*[tw] OR subdural postconcussion*[tw] OR subdural post-concussion*[tw] OR subdural hemorrhag*[tw] OR subdural haemorrhag*[tw] OR subdural hematom*[tw] OR subdural bleed*[tw] OR subdural penetrat*[tw] OR subdural nonpenetrat*[tw] OR subdural non-penetrat*[tw]	11639	9:41:25
#35	Search dural injur*[tw] OR dural traum*[tw] OR dural concussion*[tw] OR dural postconcussion*[tw] OR dural post-concussion*[tw] OR dural hemorrhag*[tw] OR dural haemorrhag*[tw] OR dural hematom*[tw] OR dural bleed*[tw] OR dural penetrat*[tw] OR dural nonpenetrat*[tw] OR dural non-penetrat*[tw]	275	9:41:02
#34	Search Putaminal injur*[tw] OR Putaminal traum*[tw] OR Putaminal concussion*[tw] OR Putaminal postconcussion*[tw] OR Putaminal post-concussion*[tw] OR Putaminal hemorrhag*[tw] OR Putaminal haemorrhag*[tw] OR Putaminal hematom*[tw] OR Putaminal bleed*[tw] OR Putaminal penetrat*[tw] OR Putaminal nonpenetrat*[tw] OR Putaminal non-penetrat*[tw]	391	9:40:39

#33	Search putamen injur*[tw] OR putamen traum*[tw] OR putamen concussion*[tw] OR putamen postconcussion*[tw] OR putamen post-concussion*[tw] OR putamen hemorrhag*[tw] OR putamen haemorrhag*[tw] OR putamen hematom*[tw] OR putamen bleed*[tw] OR putamen penetrat*[tw] OR putamen nonpenetrat*[tw] OR putamen non-penetrat*[tw]	879	9:32:55
#32	Search pontine injur*[tw] OR pontine traum*[tw] OR pontine concussion*[tw] OR pontine postconcussion*[tw] OR pontine post-concussion*[tw] OR pontine hemorrhag*[tw] OR pontine haemorrhag*[tw] OR pontine hematom*[tw] OR pontine bleed*[tw] OR pontine penetrat*[tw] OR pontine nonpenetrat*[tw] OR pontine non-penetrat*[tw]	962	9:32:37
#31	Search head injur*[tw] OR head traum*[tw] OR head concussion*[tw] OR head postconcussion*[tw] OR head post-concussion*[tw] OR head hemorrhag*[tw] OR head haemorrhag*[tw] OR head hematom*[tw] OR head bleed*[tw] OR head penetrat*[tw] OR head nonpenetrat*[tw] OR head non-penetrat*[tw]	32437	9:32:19
#30	Search Cerebellum injur*[tw] OR Cerebellum traum*[tw] OR Cerebellum concussion*[tw] OR Cerebellum postconcussion*[tw] OR Cerebellum post-concussion*[tw] OR Cerebellum hemorrhag*[tw] OR Cerebellum haemorrhag*[tw] OR Cerebellum hematom*[tw] OR Cerebellum bleed*[tw] OR Cerebellum penetrat*[tw] OR Cerebellum nonpenetrat*[tw] OR Cerebellum non-penetrat*[tw]	4288	9:32:06
#29	Search cerebellar injur*[tw] OR cerebellar traum*[tw] OR cerebellar concussion*[tw] OR cerebellar postconcussion*[tw] OR cerebellar post-concussion*[tw] OR cerebellar hemorrhag*[tw] OR cerebellar haemorrhag*[tw] OR cerebellar hematom*[tw] OR cerebellar bleed*[tw] OR cerebellar penetrat*[tw] OR cerebellar nonpenetrat*[tw] OR cerebellar non-penetrat*[tw]	1068	9:31:54
#28	Search skull injur*[tw] OR skull traum*[tw] OR skull concussion*[tw] OR skull postconcussion*[tw] OR skull post-concussion*[tw] OR skull hemorrhag*[tw] OR skull haemorrhag*[tw] OR skull hematom*[tw] OR skull bleed*[tw] OR skull penetrat*[tw] OR skull nonpenetrat*[tw] OR skull non-penetrat*[tw]	9927	9:31:41
#27	Search intra-cerebral injur*[tw] OR intra-cerebral traum*[tw] OR intra-cerebral concussion*[tw] OR intra-cerebral postconcussion*[tw] OR intra-cerebral post-concussion*[tw] OR intra-cerebral hemorrhag*[tw] OR intra-cerebral haemorrhag*[tw] OR intra-cerebral hematom*[tw] OR intra-cerebral bleed*[tw] OR intra-cerebral penetrat*[tw] OR intra-cerebral nonpenetrat*[tw] OR intra-cerebral non-penetrat*[tw]	191	9:31:29
#26	Search intracerebral injur*[tw] OR intracerebral traum*[tw] OR intracerebral concussion*[tw] OR intracerebral postconcussion*[tw] OR intracerebral post-concussion*[tw] OR intracerebral hemorrhag*[tw] OR intracerebral haemorrhag*[tw] OR intracerebral hematom*[tw] OR intracerebral bleed*[tw] OR intracerebral penetrat*[tw] OR intracerebral nonpenetrat*[tw] OR intracerebral non-penetrat*[tw]	13499	9:31:16

#25	Search intra-cranial injur*[tw] OR intra-cranial traum*[tw] OR intra-cranial concussion*[tw] OR intra-cranial postconcussion*[tw] OR intra-cranial post-concussion*[tw] OR intra-cranial hemorrhag*[tw] OR intra-cranial haemorrhag*[tw] OR intra-cranial hematom*[tw] OR intra-cranial bleed*[tw] OR intra-cranial penetrat*[tw] OR intra-cranial nonpenetrat*[tw] OR intra-cranial non-penetrat*[tw]	110	9:30:58
#24	Search intracranial injur*[tw] OR intracranial traum*[tw] OR intracranial concussion*[tw] OR intracranial postconcussion*[tw] OR intracranial post-concussion*[tw] OR intracranial hemorrhag*[tw] OR intracranial haemorrhag*[tw] OR intracranial hematom*[tw] OR intracranial bleed*[tw] OR intracranial penetrat*[tw] OR intracranial nonpenetrat*[tw] OR intracranial non-penetrat*[tw]	16804	9:30:47
#23	Search cranial injur*[tw] OR cranial traum*[tw] OR cranial concussion*[tw] OR cranial postconcussion*[tw] OR cranial post-concussion*[tw] OR cranial hemorrhag*[tw] OR cranial haemorrhag*[tw] OR cranial hematom*[tw] OR cranial bleed*[tw] OR cranial penetrat*[tw] OR cranial nonpenetrat*[tw] OR cranial non-penetrat*[tw]	3372	9:30:34
#22	Search encephalon injur*[tw] OR encephalon trauma*[tw] OR encephalon concussion*[tw] OR encephalon postconcussion*[tw] OR encephalon post-concussion*[tw] OR encephalon hemorrhag*[tw] OR encephalon haemorrhag*[tw] OR encephalon hematoma*[tw] OR encephalon bleed*[tw] OR encephalon penetrat*[tw] OR encephalon nonpenetrat*[tw] OR encephalon non-penetrat*[tw]	118061	9:30:15
#21	Search TBI* OR Brain injur*[tw] OR brains injur*[tw] OR Brain trauma*[tw] OR Brain concussion*[tw] OR Brain postconcussion*[tw] OR Brain post-concussion*[tw] OR Brain hemorrhag*[tw] OR Brain haemorrhag*[tw] OR Brain hematoma[tw] OR Brain hematom*[tw] OR Brain bleed*[tw] OR Brain penetrat*[tw] OR Brain nonpenetrat*[tw] OR Brain non-penetrat*[tw]	149189	9:29:55
#20	Search Brain Injuries [mesh]	57395	9:29:33
#19	Search military* OR veteran* OR army force*[tw] OR armed force* [tw] OR armies OR navy OR marine* OR soldier* OR OIF OR OEF OR deployment OR service member*[tw] OR operation freedom*[tw] OR operations freedom*[tw] OR operation Dawn*[tw] OR operations Dawn*[tw] OR war OR wars OR afghan* OR Iraq* OR gulf	477092	9:29:13
#18	Search war exposure[mesh]	204	9:28:59
#17	Search iraq war, 2003-2011[mesh]	2413	9:28:47
#16	Search gulf war[mesh]	375	9:28:35
#15	Search afghan campaign 2001-[mesh]	2119	9:28:24
#14	Search hospitals, veterans[mesh]	6155	9:28:10
#13	Search hospitals, military[mesh]	4919	9:28:03
#12	Search Military Personnel[mesh]	34750	9:27:42
#11	Search Military Facilities[mesh]	146	9:27:30

#10	Search Naval Medicine[mesh]	9353	9:27:18
#9	Search "United States Department of Defense"[mesh]	242	9:26:39
#8	Search Military Medicine[mesh]	28222	9:26:02
#7	Search Veterans Disability Claims[mesh]	271	9:25:32
#6	Search Veterans[mesh]	12574	9:24:16
#5	Search Veterans Health[mesh]	717	9:23:53
#4	Search "United States Department of Veterans Affairs"[mesh]	6219	9:23:41
#3	Search blast* OR explosion* OR detonat*	85385	9:23:22
#2	Search Explosions[mesh]	3617	9:23:06
#1	Search Blast Injuries[mesh]	3671	9:22:51

(TITLE-ABS-KEY (blast* OR explosion* OR detonat*))

AND

(TITLE-ABS-KEY (military* OR veteran* OR ((army or armed) W/3 force*) OR armies OR navy OR marine* OR soldier* OR OIF OR OEF OR deployment OR (service adj3 W/3 member*) OR (operation* W/3 (freedom OR Dawn)) OR war OR wars OR afghan* OR Iraq* OR gulf))

AND

(TITLE-ABS-KEY ((brain* OR encephalon* OR cranium OR cranio* OR cranial OR intracrani* OR intra-crani* OR skull* OR cerebral OR cerebell* OR head OR ventric* OR pontine OR putamin* OR dura* OR subdura* OR sub-dura* OR (supra OR extra W/2 dura*) OR supradura* OR epidura* OR epi-dura* OR arachnoid* OR sub-arachnoid* OR subarachnoid* OR (intra W/2 arachnoid*)) W/3 (injur* OR trauma* OR concussion* OR post-concussion* OR hemorrhag* OR haemorrhag* OR hematom* OR haematom* OR bleed* OR penetrat* OR (non W/2 penetrat*) OR edema* OR oedema* OR fracture* OR aneurysm* OR pressur*))) OR (TITLE-ABS-KEY (pneumocephal* OR pneumo-cephal* OR aerocele* OR airocele* OR pneumocyst* OR pneumo-cyst* OR ((csf OR cerebrospinal) W/3 (leak* OR otorrhea* OR rhinorrhea*)) OR contrecoup* OR (contre W/3 coup*))) OR (TITLE-ABS-KEY (neurosurg* OR neuro-surg* OR craniotom* OR craniectom* OR trepanation* OR trepanning* OR trephination* OR trephining*))

Search Name:

Date Run: 02/04/17 10:51:51.604

Description:

ID	Search	Hits
#1	MeSH descriptor: [Blast Injuries] explode all trees	13
#2	MeSH descriptor: [Explosions] explode all trees	5
#3	blast* or explosion* or detonat*:ti,ab,kw (Word variations have been searched)	1827
#4	MeSH descriptor: [United States Department of Veterans Affairs] explode all trees	254
#5	MeSH descriptor: [Veterans Health] explode all trees	26
#6	MeSH descriptor: [Veterans] explode all trees	691
#7	MeSH descriptor: [Veterans Disability Claims] explode all trees	3
#8	MeSH descriptor: [Military Medicine] explode all trees	167
#9	MeSH descriptor: [United States Department of Defense] explode all trees	4
#10	MeSH descriptor: [Naval Medicine] explode all trees	56
#11	MeSH descriptor: [Military Facilities] explode all trees	44
#12	MeSH descriptor: [Military Personnel] explode all trees	759
#13	MeSH descriptor: [Hospitals, Military] explode all trees	45
#14	MeSH descriptor: [Hospitals, Veterans] explode all trees	309
#15	MeSH descriptor: [Afghan Campaign 2001-] explode all trees	42
#16	MeSH descriptor: [Gulf War] explode all trees	10
#17	MeSH descriptor: [Iraq War, 2003-2011] explode all trees	46
#18	MeSH descriptor: [War Exposure] explode all trees	3
#19	military* or veteran* or ((army or armed) near/3 force*) or armies or navy or marine* or soldier* or OIF or OEF or deployment or (service near/3 member*) or (operation* near/3 (freedom or Dawn)) or war or wars or afghan* or Iraq* or gulf:ti,ab,kw (Word variations have been searched)	6835

#20 MeSH descriptor: [Brain Injuries] explode all trees 1382

#21 MeSH descriptor: [Neurosurgery] explode all trees 101

#22 MeSH descriptor: [Craniotomy] explode all trees411

#23 MeSH descriptor: [Decompressive Craniectomy] explode all trees 22

#24 MeSH descriptor: [Trephining] explode all trees 6

#25 MeSH descriptor: [Brain Injury, Chronic] explode all trees 26

#26 MeSH descriptor: [Brain Edema] explode all trees 153

#27 MeSH descriptor: [Brain Concussion] explode all trees 113

#28 MeSH descriptor: [Contrecoup Injury] explode all trees 0

#29 MeSH descriptor: [Post-Concussion Syndrome] explode all trees 33

#30 MeSH descriptor: [Brain Hemorrhage, Traumatic] explode all trees 13

#31 MeSH descriptor: [Brain Stem Hemorrhage, Traumatic] explode all trees 0

#32 MeSH descriptor: [Pneumocephalus] explode all trees 6

#33 MeSH descriptor: [Intracranial Hemorrhages] explode all trees 1596

#34 MeSH descriptor: [Cerebral Hemorrhage] explode all trees 807

#35 MeSH descriptor: [Putaminal Hemorrhage] explode all trees 6

#36 MeSH descriptor: [Intracranial Hemorrhage, Hypertensive] explode all trees 22

#37 MeSH descriptor: [Intracranial Hemorrhage, Traumatic] explode all trees103

#38 MeSH descriptor: [Hematoma, Epidural, Cranial] explode all trees 12

#39 MeSH descriptor: [Hematoma, Subdural, Acute] explode all trees 4

#40 MeSH descriptor: [Hematoma, Subdural] explode all trees 65

#41 MeSH descriptor: [Hematoma, Subdural, Chronic] explode all trees 33

#42 MeSH descriptor: [Hematoma, Subdural, Intracranial] explode all trees 5

#43 MeSH descriptor: [Subarachnoid Hemorrhage, Traumatic] explode all trees 4

#44 MeSH descriptor: [Pituitary Apoplexy] explode all trees 1

#45 MeSH descriptor: [Subarachnoid Hemorrhage] explode all trees 512

#46 MeSH descriptor: [Subarachnoid Hemorrhage, Traumatic] explode all trees 4

#47 MeSH descriptor: [Intracranial Hypertension] explode all trees 153

#48 MeSH descriptor: [Trauma, Nervous System] explode all trees 3476

#49 MeSH descriptor: [Craniocerebral Trauma] explode all trees 2415

#50 MeSH descriptor: [Cerebrospinal Fluid Leak] explode all trees 53

#51 MeSH descriptor: [Cerebrospinal Fluid Otorrhea] explode all trees 32

#52 MeSH descriptor: [Cerebrospinal Fluid Rhinorrhea] explode all trees 24

#53 MeSH descriptor: [Coma, Post-Head Injury] explode all trees 5

#54 MeSH descriptor: [Cranial Nerve Injuries] explode all trees 120

#55 MeSH descriptor: [Head Injuries, Closed] explode all trees 190

#56 MeSH descriptor: [Head Injuries, Penetrating] explode all trees 1

#57 MeSH descriptor: [Skull Fractures] explode all trees 224

#58 MeSH descriptor: [Skull Fracture, Basilar] explode all trees 2

#59 MeSH descriptor: [Cerebrovascular Trauma] explode all trees 36

#60 TBI* or mTBI*:ti,ab,kw (Word variations have been searched) 1375

#61 (brain* or encephalon* or cranium or cranio* or cranial or intracrani* or intra-crani* or skull* or cerebral or cerebell* or head or ventric* or hemispher* or pontine or putamin* or dura* or subdura* or sub-dura* or (supra near/2 dura*) or supradura* or epidura* or epi-dura* or arachnoid* or sub-arachnoid* or subarachnoid* or (intra near/2 arachnoid*)) near/3 (injur* or trauma* or concussion* or post-concussion* or hemorrhag* or haemorrhag* or hematom* or haematom* or bleed* or penetrat* or (non near/2 penetrat*) or edema* or oedema* or fracture* or aneurysm* or pressur* or lesion* or swell* or contusion*):ti,ab,kw (Word variations have been searched) 15792

#62 neurosurg* or neuro-surg* or craniotom* or craniectom* or trepanation* or trepanning* or trephination* or trephining*:ti,ab,kw (Word variations have been searched) 2790

#63 pneumocephal* or pneumo-cephal* or airocele* or aerocele* or pneumocyst* or pneumo-cyst* or ((CSF or cerebrospinal) near/3 (leak* or otorrhea* or rhinorrhea*)) or contrecoup* or countrecoup* or (contre or countre near/3 coup*):ti,ab,kw (Word variations have been searched) 788

#64 #1 or #2 or #3 1827

#65 #4 or #5 or #6 or #7 or #8 or #9 or #10 or #11 or #12 or #13 or #14 or #15 or #16 or #17 or #18 or #19 6860

#66 #20 or #21 or #22 or #23 or #24 or #25 or #26 or #27 or #28 or #29 or #30 or #31 or #32 or #33 or #34 or #35 or #36 or #37 or #38 or #39 or #40 or #41 or #42 or #43 or #44 or #45 or #46 or #47 or #48 or #49 or #50 or #51 or #52 or #53 or #54 or #55 or #56 or #57 or #58 or #59 or #60 or #61 or #62 or #63 20351

#67 #64 and #65 and #66 18

#1	189,132	TS = (blast* or explosion* or detonat*)<br>Indexes=SCI-EXPANDED, SSCI, A&HCI, CPCI-S, CPCI-SSH, BKCI-S, BKCI-SSH, ESCI Timespan=All years
#2	849,151	TS = (military* or veteran* or ((army or armed) NEAR/3 force*) or armies or navy or marine* or soldier* or OIF or OEF or deployment or (service NEAR/3 member*) or (operation* NEAR/3 (freedom or Dawn)) or war or wars or afghan* or Iraq* or gulf)<br>Indexes=SCI-EXPANDED, SSCI, A&HCI, CPCI-S, CPCI-SSH, BKCI-S, BKCI-SSH, ESCI Timespan=All years
#3	199,873	TS = (TBI* or (brain* or encephalon* or cranium or cranio* or cranial or intracrani* or intra-crani* or skull* or cerebral or cerebell* or head or ventric* or pontine or putamin* or dura* or subdura* or sub-dura* or (supra or extra NEAR/2 dura*) or supradura* or epidura* or epi-dura* or arachnoid* or sub-arachnoid* or subarachnoid* or (intra NEAR/2 arachnoid*)) NEAR/3 (injur* or trauma* or concussion* or post-concussion* or h?emorrhag* or h?ematom* or bleed* or penetrat* or (non NEAR/2 penetrat*) or edema* or oedema* or fracture* or anyeurysm* or pressur*))<br>Indexes=SCI-EXPANDED, SSCI, A&HCI, CPCI-S, CPCI-SSH, BKCI-S, BKCI-SSH, ESCI Timespan=All years
#4	47,652	TS = (neurosurg* or neuro-surg* or craniotom* or craniectom* or trepanation* or trepanning* or trephination* or trephining*)<br>Indexes=SCI-EXPANDED, SSCI, A&HCI, CPCI-S, CPCI-SSH, BKCI-S, BKCI-SSH, ESCI Timespan=All years
#5	19,331	TS = (pneumocephal* or pneumo-cephal* or a?rocele* or pneumocyst* or pneumo-cyst* or ((CSF or cerebrospinal) NEAR/3 (leak* or otorrhea* or rhinorrhea*)) or co?ntrecoup* or (co?ntre NEAR/3 coup*))<br>Indexes=SCI-EXPANDED, SSCI, A&HCI, CPCI-S, CPCI-SSH, BKCI-S, BKCI-SSH, ESCI Timespan=All years
#6	256,563	#5 OR #4 OR #3<br>Indexes=SCI-EXPANDED, SSCI, A&HCI, CPCI-S, CPCI-SSH, BKCI-S, BKCI-SSH, ESCI Timespan=All years
#7	790	#6 AND #2 AND #1<br>Indexes=SCI-EXPANDED, SSCI, A&HCI, CPCI-S, CPCI-SSH, BKCI-S, BKCI-SSH, ESCI Timespan=All years

SourcesEmbase, MEDLINE
Query'blast injury'/exp OR 'explosion'/exp OR blast*:ab,ti OR explosion*:ab,ti OR detonat*:ab,ti AND 'veterans health'/exp OR 'veteran'/exp OR 'military family'/exp OR 'government'/exp OR 'military medicine'/exp OR 'military phenomena'/exp OR 'soldier'/exp OR 'war'/exp OR military*:ab,ti OR veteran*:ab,ti OR 'army force*':ab,ti OR 'armed force*':ab,ti OR armies:ab,ti OR navy:ab,ti OR marine*:ab,ti OR soldier*:ab,ti OR oif*:ab,ti OR oef*:ab,ti OR deployment*:ab,ti OR 'service member*':ab,ti OR 'operation freedom*':ab,ti AND 'operation dawn*':ab,ti OR war:ab,ti AND tbi*:ab,ti OR mtbi*:ab,ti OR ((brain* OR encephalon* OR cranium OR cranio* OR cranial OR intracrani* OR 'intra crani*' OR skull* OR cerebral OR cerebell* OR head OR ventric* OR hemispher* OR pontine OR putamin* OR dura* OR subdura* OR 'sub dura*' OR extradural* OR supradura* OR epidura* OR 'epi dura*' OR arachnoid* OR 'sub arachnoid*' OR subarachnoid* OR 'intra arachnoid*' OR intraarachnoid*) NEAR/3 (injur* OR trauma* OR concussion* OR 'post concussion*' OR hemorrhag* OR haemorrhag* OR hematom* OR haematom* OR bleed* OR penetrat* OR 'nonpenetrat*' OR nonpenetrat* OR edema* OR oedema* OR fracture* OR aneurysm* OR pressur* OR lesion* OR swell* OR contusion*)):ab,ti OR pneumocephal*:ab,ti OR 'pneumo cephal*':ab,ti OR aerocele*:ab,ti OR airocele*:ab,ti OR pneumocyst*:ab,ti OR 'pneumo cyst*':ab,ti OR ((csf OR cerebrospinal) NEAR/3 (leak* OR otorrhea* OR rhinorrhea*)):ab,ti OR contrecoup*:ab,ti OR countrecoup*:ab,ti OR 'contre coup*':ab,ti OR 'countre coup*':ab,ti OR neurosurg*:ab,ti OR 'neuro surg*':ab,ti AND craniotom*:ab,ti OR craniectom*:ab,ti OR trepanation*:ab,ti OR trepanning*:ab,ti OR trephination*:ab,ti OR trephining*:ab,ti OR 'brain injury'/exp OR 'brain edema'/exp OR 'contrecoup injury'/exp OR 'brain hemorrhage'/exp OR 'intracranial hypertension'/exp OR 'liquorrhea'/exp OR 'head injury'/exp OR 'coma'/exp
